# Tracking Reactivation of Location Information during Memory Strategies: Insights from Eye Movements

**DOI:** 10.5334/joc.449

**Published:** 2025-07-02

**Authors:** Ruhi Bhanap, Lea M. Bartsch, Agnes Rosner

**Affiliations:** 1 University of Zurich, Switzerland; 2 Leibniz University Hannover, Germany

**Keywords:** Working Memory, Eye Movements, Strategies, Rehearsal, Visual Imagery

## Abstract

Memory strategies such as visual imagery and rehearsal are widely reported by participants as means to enhance recall. Their underlying mechanisms are thought to differ. Visual imagery is believed to engage both visual and spatial aspects of memoranda, while rehearsal is thought to reactivate only the item-specific information, excluding spatial information. In this study, we employed the Looking at Nothing (LAN) effect – in which individuals make eye movements towards the original location of the memorized item during retrieval – to investigate the reactivation of spatial location in both visual imagery and rehearsal. Our findings demonstrate that LAN occurs with both strategies, indicating that spatial information is reactivated during rehearsal as well. Notably, we observed higher immediate as well as delayed memory performance with visual imagery compared to rehearsal. However, the amount of LAN observed for both these strategies remained the same. To further explore whether these differences in the amount of LAN and memory performance were driven by a modulation of the strength of long-term memory (LTM) traces we introduced proactive interference (PI) in a second experiment. PI is known to impact LTM traces, while leaving working memory (WM) intact. While PI led to a decline in WM for visual imagery, the amount of LAN remained the same. These results indicate that visual imagery and rehearsal both reactivate location information and additionally, visual imagery drives eye movements and memory benefits through distinct mechanisms.

When having to remember new information, such as a list of vocabulary, people have been shown to engage in strategies to help improve memory performance (Bartsch et al., 2024; Mazuryk & Lockhart, 1974; Shaughnessy, 1981). These can include strategies such as articulatory rehearsal or elaboration. Articulatory rehearsal has been defined as the process during which people repeat or re-encode the to be memorized verbal information in their heads or aloud (Baddeley, 1996). Within the context of Elaboration, it has been proposed that people engage in processes that enrich memory representations by linking them to previously formed semantic associations (Craik & Tulving, 1975). Elaboration includes visual imagery, wherein people form a mental image linking the content and locations of information that they need to remember (Peterson et al., 1977).

## Strategies and Memory Performance

Previous work in the field of memory has investigated whether these strategies help in improving memory performance – that is working memory (WM) as well as long-term memory (LTM). WM is a system that stores limited information for current cognitive processing and has been studied using immediate memory tests (Cowan, 2017). LTM is a system with virtually unlimited capacity that stores information for a long duration (Tulving, 1983). Information encoded in WM can be transferred to LTM under certain conditions. Early theories suggest that rehearsal and other memory control processes facilitate this transfer (Atkinson & Shiffrin, 1968). Current theories propose that WM generally functions as an activated part of LTM, in which representations are in a heightened state of accessibility (Cowan, 1999; Oberauer, 2002). Recent research suggests that people draw on episodic traces also in an immediate test when WM capacity is exceeded (Bartsch & Oberauer, 2023) Building on these perspectives, the current study assumes that WM and LTM function as embedded systems, facilitating information transfer while allowing LTM to support immediate retrieval as more information is encoded.

Research on memory strategies has produced varying outcomes, particularly regarding the effects of rehearsal and visual imagery on WM and LTM retrieval. Tan and Ward (2008) found that slower word presentation rates allowed for more rehearsal, which correlated with better immediate recall, suggesting a beneficial effect of rehearsal for WM retrieval. However, Souza and Oberauer (2018, 2020) found no difference between articulatory rehearsal and articulatory suppression instructions, indicating that rehearsal may not always enhance WM performance. This discrepancy might be due to differences in methodology, with Tan and Ward (2008) using a correlational design while Souza and Oberauer (2018) experimentally manipulated rehearsal by providing participants rehearsal schedules and then comparing it to a condition of articulatory suppression (AS) – which is assumed to (fully) block any rehearsal. As for the effect of rehearsal on LTM, Greene (1987) summarized studies on the effect of rehearsal on recall and recognition, wherein he reports that longer rehearsal duration intervals can lead to a consistent benefit for recognition tests. Others argue and have shown that only elaborative rehearsal affects retrieval from LTM. Overall, most findings speak towards a limited benefit of rehearsal on delayed memory tests (Mazuryk & Lockhart, 1974; Shaughnessy, 1981; Craik and Lockhart 1972).

Visual imagery, in contrast, has consistently been shown to improve LTM (e.g. Dunlosky & Hertzog, 2001). Studies by Bartsch et al. (2018, 2019) demonstrated that when participants were instructed to form visual images of lists of words, their performance on delayed memory tests improved significantly compared to when they were simply asked to repeatedly read the words. Critically, these studies also found that visual imagery did not enhance immediate recall, highlighting its specific advantage for long-term retention. A recent study by Bartsch and colleagues (2024) further investigated the effects of visual imagery and rehearsal on memory. They found that both strategies produced similar immediate memory performance, but visual imagery led to stronger LTM traces, resulting in better delayed recall than rehearsal.

Considering the differential effects that rehearsal and visual imagery have on WM and LTM, their underlying mechanisms may also not be the same. A prevalent theory in the field proposes that rehearsal and visual imagery engage two different components of WM (Baddeley, 1996): Rehearsal engages the phonological loop, which is responsible for processing and maintaining acoustic or verbal information. Visual imagery engages the visuo-spatial sketchpad, which is responsible for processing and maintaining visual and spatial information. The distinction between which component is engaged and to what extent during rehearsal and visual imagery raises the question about what kind of information is activated during each of them. If rehearsal only engages the phonological loop, it will only activate the verbal information as activating the spatial information would require engaging the visuo-spatial sketchpad, too. By contrast, according to Baddeley’s theory, visual imagery engages the visuo-spatial sketchpad and therefore activates both visual and spatial information. In other words, rehearsal should only activate item information and visual imagery should activate both item and location information. Similarly, the Time-Based Resource Sharing Model (TBRS; Camos & Barrouillet, 2014) proposes that articulatory rehearsal engages a phonological buffer which is in charge of maintaining verbal information. They also include a visuo-spatial buffer as part of the model, yet it does not play a role in articulatory rehearsal. Thus, the idea that rehearsal does not activate the spatial component of WM while visual imagery does, has been the popular view in explaining the underlying mechanisms of these two maintenance strategies.

Evidence for part of this claim comes from Pearson and colleagues (1999) who showed that the visuo-spatial sketchpad indeed is necessary for spatial manipulation of visual images during mental synthesis. They showed that if participants performed a spatial tapping task alongside the mental synthesis, it can interfere with the process – thus, providing evidence that visual and spatial components of WM are recruited for creating visual images. Other work has shown that location information does get activated when participants engage in rehearsal of spatial locations (Tremblay et al., 2006), however, no study so far has investigated if the same happens for rehearsal of verbal material.

Therefore, to date, it remains untested whether the differences in memory performance between rehearsal and visual imagery are caused by differences in the extent to which location information is activated during each of them. Before speculating about how exactly location information is used to enhance memory in different strategies, a necessary first step is to directly test the hypothesis that location information is not activated during rehearsal of verbal material whereas it is for visual imagery within a single study. This was the first goal of the present study.

## Eye Movements and Memory Retrieval

To investigate the theoretical question of whether location information is only activated by visual imagery and not by rehearsal, we need a tool that allows us to assess the degree of activation of location information. Recent work suggests that eye movements can reflect whether location information is activated. Looking at Nothing (LAN) refers to a behavior wherein during retrieval, people tend to look back at locations where information was previously presented (Richardson & Spivey, 2000; Wynn et al., 2022). In a typical LAN paradigm, people encode visual or verbal information presented at different locations on the screen. Usually, this is followed by a retrieval phase where item information is removed while the location information remains on the screen. It has been shown that participants tend to look at the empty spatial location of the to-be retrieved information during this time. LAN has been explained as the result of activations in a spatial priority map, which is shared by the focus of attention (FoA) and visual attention. The shared priority map represents the item-context bindings held in WM and the extent to which each binding is activated through the contributions of the FoA and visual attention. The FoA is a selection mechanism within WM which selects a single item-context binding that is required for the current cognitive process. The activations as part of the shared priority map feed into the eye movement system (Hedge & Leonards, 2013; Theeuwes et al., 2009) and eye movements are directed towards the location that is highly activated. Thus, LAN has been argued to reflect the binding selected in the FoA and it is expressed as a shift of attention towards the associated location (Bhanap et al., 2025).

Most of the previous work – but not all (see Bhanap et al., 2025; Hoover & Richardson, 2008; Richardson & Spivey, 2000; Scholz et al., 2016) – has investigated LAN in the context of visual imagery instructions. In a study by Marterelli and Mast (2013), participants were presented with pictures and were asked to generate and inspect mental images. They showed LAN for both generation and inspection of mental images, with higher LAN for the latter. Another study by Johansson and colleagues (2012) presented participants with audio or visual images of scenes followed by the instruction to imagine and narrate the scene. The study reported that participants recreated the gaze patterns that were present during encoding of the scenes.

These results have been explained by the visual buffer model of Kosslyn et al. (2006) which states that creating and inspecting visual images leads to internal shifts of attention and these are connected to the eye movement system which is reflected in LAN. An alternative explanation of the LAN and imagery connection is the Enactive Theory of Visual Imagery (Thomas, 2009). According to this theory, visual imagery is not only used to retrieve information from memory, but it is rather about actively creating visual experiences. That is, people are supposedly enacting or mimicking the processes engaged when perceiving the event. Thus, during LAN, we observe eye movements that resemble the ones that were made at encoding, but they are not a result of retrieval of the memory but rather an act of perception. The current state of research favors the idea that LAN is observed in the service of building and inspecting mental images (Chiquet et al., 2021; Gurtner et al., 2021; Johansson et al., 2012; Martarelli & Mast, 2013; Umar et al., 2021). Past research on LAN has not exclusively focused on visual imagery involving visual scenes to memorize. Several studies have examined LAN using verbal information (Bhanap et al., 2024; Hoover & Richardson, 2008; Richardson & Spivey, 2000; Scholz et al., 2016). Notably, in these studies, location information was irrelevant, requiring participants to maintain only verbal information. Despite this, LAN is still observed. A possible reason for this could be that prior research indicates that participants often report engaging in visual imagery even when not explicitly instructed to use a particular strategy (Bartsch et al., 2024). This spontaneous use of visual imagery could contribute to the observed LAN in such studies.

Support for this comes from a study by Kumcu and Thompson (2020), who investigated LAN by presenting words under two conditions: high difficulty (low imageability) and low difficulty (high imageability). They found higher LAN for less imageable words. They explained their findings as follows: LAN engages visual imagery processes and that for less imageable words, LAN compensates for the imagery deficit, resulting in greater LAN in these conditions. They suggest that LAN inherently involves visual imagery processes, even when participants are tasked with maintaining verbal information alone. Critically, none of the above studies which investigated the connection of visual imagery and LAN – neither on visual nor verbal material – have any condition of comparison, in which LAN is either actively prevented or another strategy is instructed to see if the latter claim holds. One study by Rosner, Schaffner and von Helversen (2022), instructed participants to use visual imagery in a visual categorization task and reported higher LAN as compared to a baseline condition. As it was a decision-making task and not a memory task, the baseline condition included the instruction to use the knowledge that participants had learned in a separate session before the task. So also here, the occurrence of LAN was not compared between different maintenance strategies. Thus, the second goal of the study was to investigate what drives LAN under different strategies.

## The present study

The aim of the present study is to bring together the above lines of research, which is reflected in two goals: First, we investigate what information people reactivate during the engagement in strategies such as rehearsal and visual imagery – which to date is unclear. Theories propose a dichotomy between the activation of mere item information (verbal, during rehearsal) and spatial information (visual imagery; Baddeley, 1996; Camos & Barrouillet, 2014). If this distinction holds, we expect to observe higher LAN for visual imagery compared to rehearsal. Alternatively, Ferreira and colleagues (2008) suggest that when the information is encoded, all aspects of it – meaning the visual, verbal, and spatial information – form an integrated representation. After that, when the visual or verbal information is retrieved, it also leads to the retrieval of spatial information accompanied by eye movements to the stored spatial locations. Thus, this alternative idea would assume that in case participants engage in the rehearsal of verbal information, this would also lead to activating location information.

Our second goal is to understand the mechanisms that lead to LAN when people engage in different maintenance strategies. To investigate this, we formulate two hypotheses based on research on eye movements, particularly in relation to LAN. Previous studies suggest that creating and inspecting visual images leads to internal shifts of attention (e.g., Martarelli & Mast, 2013), which in turn leads to LAN. However, it remains unclear whether these eye movements are exclusive to visual imagery or if they also occur under other strategy conditions.

The first hypothesis, which we refer to as the Imagery LAN Hypothesis, predicts that LAN will be more pronounced during visual imagery than in other strategies. This expectation is grounded in prior research, which has not only observed LAN in response to visual imagery (e.g., Johansson et al., 2012) but also reported similar effects for verbal materials (e.g., Richardson & Spivey, 2000). Some studies have interpreted this as evidence that LAN is specifically tied to the visual processing required for mental imagery. However, the existing literature is limited in that previous studies have primarily focused on visual imagery instructions without systematically comparing LAN across different strategy conditions or establishing a proper baseline.

This hypothesis also connects to our first research goal and to theories of WM, which propose that rehearsal and visual imagery rely on separate buffers. If this assumption holds, only visual imagery should lead to the reactivation of spatial information, manifesting as LAN behavior. Our study allows us to test these predictions within a single investigation, offering new insights into both memory processes and LAN mechanisms.

There is a second, alternative explanation for the observation of LAN in a working memory task under different strategy instructions: A well-established finding in WM research is that accuracy on immediate memory tests declines as the number of elements in the memory set increases. This decline suggests a corresponding decrease in the strength of memory traces with larger set sizes (Crannell & Parrish, 1957; Luck & Vogel, 1997; Shah & Miyake, 1996). We hereby consider that performance in a given test situation is determined by some combination of quality and availability of a memory representation, which we summarize under the term “memory strength”. If LAN is linked to the strength of the memory representation rather than the strategy, its modulation will be determined by the trace’s strength. Support for this comes from two lines of thought. For one, there are studies showing higher LAN for correct memory retrieval (e.g., Laeng et al., 2014; Laeng & Teodorescu, 2002; Scholz et al., 2016), which is assumed to result from higher memory activation. Second, LAN has been intensively used to study decision making and reasoning processes (e.g., Renkewitz & Jahn, 2012; Rosner et al., 2022; Rosner & von Helversen, 2019; Jahn & Braatz, 2014; Klichowicz et al., 2020, 2021; Scholz et al., 2017). This literature rests on the assumption that LAN measures the activation of information in memory. Indeed, an unpublished study from our lab showed that as set size increases in verbal recall, LAN decreases (Rosner, Pantoja, & Bhanap, in prep.). If LAN measures memory strength independent of the maintenance strategy, the strategy leading to the highest strength should also lead to the strongest LAN. For example, if in our paradigm, participants demonstrate the highest performance during rehearsal in the immediate test, we should also observe the strongest LAN for rehearsal. We refer to this as the Memory Strength LAN hypothesis. Additionally, this would indicate that LAN is not a reliable tool for tracking maintenance strategies directly but is instead more indicative of the strength of working memory traces.

In summary, in the present study, we aim to investigate whether LAN is observed for both rehearsal and visual imagery. To this end, we conducted Experiment 1, in which participants were instructed to engage in either rehearsal or visual imagery. Additionally, we added two control conditions. In some trials, people were instructed to use articulatory suppression, and in other trials no strategy instructions were given. In the latter baseline condition, participants were able to engage in any maintenance strategy (or none) spontaneously, which gave us an idea of their natural behavior in the task. To get an estimate of the effect of rehearsal on behavior, we added a condition of articulatory suppression, which is assumed to block the phonological loop. The WM task phase was followed by an LTM test which allowed us to investigate the typical patterns of strategy effects across WM and LTM. We recorded eye movements throughout the experiment to monitor if participants exhibit LAN during the experiment.

## Experiment 1

### Methods

#### Participants

We collected data of 49 participants in the lab. All participants were native German speakers, between the ages of 18–35 and with normal or corrected to normal vision. Participants signed an informed consent, and the experimental protocol was in accordance with the Institutional Review Board of the Psychology Institute from University of Zurich. A document is available in the supplementary materials that mentions the deviations from the preregistration based on the criteria given by Willroth and colleagues (2024).

As preregistered, participants were excluded if their performance was below chance level in the WM or LTM task. Based on this, we excluded 6 participants, 1 for below chance performance in the WM task and 5 for below chance performance in the LTM task. Additionally, we also excluded participants if they did not meet the dwell criterion for eye movement data in the WM task (as preregistered). The first step to calculate the dwell criterion was to exclude gaze data with artefacts for each participant and each trial. If the remaining data was less than 60% of the duration of the trial for more than half the trials, we excluded the participant. Accordingly, we excluded 5 participants for not meeting the dwell criterion. Finally, we excluded one participant due to an error in recording eye movement data leading to loss of data. Thus, our final sample consisted of 38 participants (M_age_ = 23.22, SD_age_ = 3.8, 25 female).

#### Materials and Procedure

An SMI Red500 system was used for eye-tracking with a sampling rate of 500 Hz. Stimuli were presented using MATLAB 2014a with a Psychophysics Toolbox 3 extension. Participants were seated 70 cm from the screen with a resolution of 1920 × 1080 pixels.

We based the current paradigm on a previous study run in our lab, in which we studied LAN behavior during WM retrieval (Bhanap et al., 2025). Here, participants were presented with three word-pairs at three different locations during encoding. During retrieval they heard two words and were asked to indicate whether both the words formed a word pair in the encoding phase (positive probes) or not (lure or new probes).

**Figure 1:**
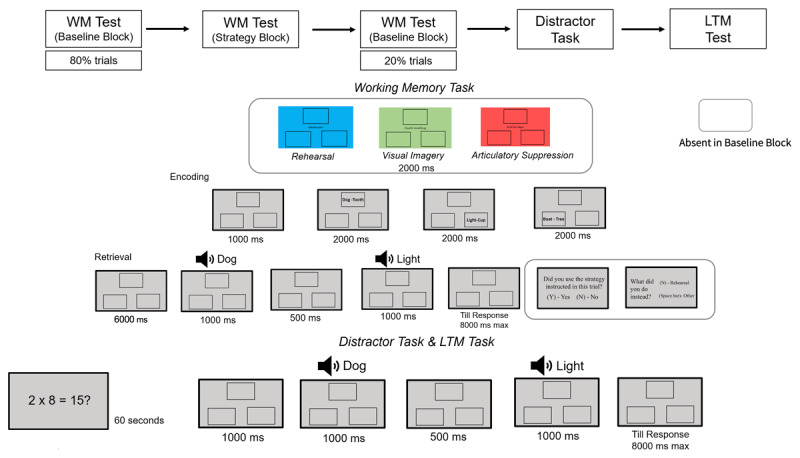
Sequence of events for Experiment 1.

The study consisted of two parts: a WM and an LTM part (see Figure 1). Each trial in the WM part was divided into an encoding phase, a retention phase, and a retrieval phase. During the encoding phase, participants were presented sequentially with three word-pairs in three different locations. The font size of the words was 22 and the size of the rectangles was 300 × 200 pixels. Each of the rectangles was presented at 10 × 6 degrees of visual angle from the center of the screen. The word pairs were presented sequentially starting from the top in a clockwise manner. Each word pair was presented for 2000 ms with an inter-stimulus-interval of 500 ms.

We manipulated the engagement of strategies through instructions, which varied from trial-to-trial. For the first 9 trials and for the last 3 trials of the WM phase, we realized the baseline condition, in which participants received no instruction and were free to engage in any strategy or do nothing. In the remaining trials, prior to the encoding phase, participants were instructed to engage in one of three different strategies: visual imagery, articulatory rehearsal, or articulatory suppression. For visual imagery, participants were asked to create a mental image of the two words in each of the word pairs. For articulatory rehearsal, they were asked to say out aloud all word pairs in cumulative fashion. For articulatory suppression, they were asked to say “babibou”. The retention interval was realized in between the encoding and retrieval phase and lasted 6 seconds.

The instructions which strategy to engage in were presented to participants prior to encoding of the list, in form of color cues accompanied with the name of the strategy at the center of the screen. Here, the screen turned into a particular color for 2 seconds, each color indicating the strategy that the participants had to engage (green: visual imagery, blue: articulatory rehearsal, red: articulatory suppression).

During the retrieval phase, participants heard two probe words with a gap of 500 ms while the empty rectangles remained on screen. The two words presented for the test were termed *first probe* and *second probe* sequentially.^1^ After participants heard the second probe, they indicated with a keypress whether the two words belonged to the same word pair (positive trials) or not (lure or new trials). In lure trials, the two probe words stemmed from different word pairs of the trial and for the new trials, a new word was presented as the second probe. Kahana’s (2002) associative symmetry theory suggests that the order of word pair presentation does not affect memory retrieval, so for both positive and lure trials, pairs were randomly presented in either forward (e.g., “Dog-Tooth”) or backward (e.g., “Tooth-Dog”) order. The trial ended after participants indicated their response or after 8 seconds had passed.

After each trial in the retrieval phase of the strategy block, participants were asked whether they indeed had engaged in the strategy they were instructed to in that trial in a two alternative forced choice manner (2-AFC) by pressing the “Y” for “yes” or “N” for “no” respectively. If they responded, “No”, they were asked to indicate what they did instead. For visual imagery, the options were between rehearsal and others, for rehearsal the options were visual imagery and others and for the articulatory suppression, the options were between visual imagery, rehearsal, and others. Participants indicated their response by pressing “Y” for visual imagery, “N” for rehearsal and “Space bar” for others. The response-option association was visible to them on screen to avoid any confusion in the associations. Additionally, to ensure compliance to rehearsal and visual imagery we recorded the speech of participants. To check for compliance with the visual imagery instruction, we assessed performance in the LTM phase (Bartsch et al., 2018). The trial ended once the participant had responded to the question/s.

Participants began the experiment with a 9-point calibration which was followed by 3 practice trials for the baseline block. For the strategy block, participants performed 2 practice trials for each of the strategy conditions.

After the WM phase, participants were presented with a distractor task for 1 minute. Here, participants were presented with math equations one after the other and they indicated with keypresses whether these equations were correct or incorrect. The structure of all the math equations was the same – a multiplication problem (e.g., “2 × 7 = 14?”). This task served to engage WM (Wilhelm et al. 2013), so that afterwards no information from the initial tasks was available in WM anymore, and the subsequent memory test relied exclusively on LTM.

For the LTM phase, participants were re-tested on the word pairs that they had seen during the WM phase. The LTM test was realized in the same way as the retrieval phase in the WM phase: participants heard two probe words interleaved by a gap of 500 ms and reported whether they recognized them as an intact word pair from the encoding phase or not. For half of the trials, the response was the same (for example, if the trial was a positive trial in the WM phase, it was a positive trial in the LTM test) and for the other half of the trials, the response was different (for example, if the trial was a positive trial in the WM phase, it was a lure or new trial in the LTM test).

During the WM phase, participants performed recalibration after every 12 trials and after the practice phase in the baseline condition and strategy condition. During the LTM test, participants were recalibrated after blocks of 32 trials.

#### Data Analysis

As dependent variable, we analyzed accuracy across strategies for the two phases (WM and LTM). We ran Generalized Linear Models in brms (Bürkner, 2017) package in R with R studio. As the accuracy for each trial was defined as 0 or 1, we assumed a Bernoulli model with a probit link function. The regression coefficients were given a Gaussian prior with a mean of 0 and a standard deviation of 1. The random effects were given a student-t distribution prior with a mean of 3, a scale of 0, and 2 degrees of freedom. The models were run separately for the WM and LTM test. We calculated d-prime from the posterior estimates of hits (correctly indicating a word pair as positive) versus false alarms (falsely indicating a lure or new probe pair as positive). We compared these estimated d-primes between each strategy condition to investigate if memory performance differed across them. For each pairwise comparison, we calculated the Bayes Factor through the Savage Dickey Density Ratio.

For the analysis of eye movements, we detected fixations with IDF Event Detector 9 (SMI, Teltow) using a peak velocity threshold of 30 degrees of visual angle per second and a minimum fixation duration of 40 ms. The main dependent variable of interest is the fixation proportions over areas of interest (AOIs). Therefore, we drew three Areas of Interest (AOI) around the three rectangles. The size of the AOIs was 860 × 440 pixels each. The fixation proportion was calculated as the summed fixation duration in one AOI divided by the summed fixation duration in all AOIs. The AOI associated with the first probe is named L1, the one associated with the second probe, only for lure trials is named L2 and the one that is not associated with any of the probes is named L0. The AOIs were contrast coded as 2 for L1 and –1 for the remaining AOIs as a similar previous study by Bhanap and colleagues (2024) only observed LAN to L1. For the fixation proportion analysis, as the values range from 0 to 1, we ran Ordered Beta Regression (OBR) Models (Kubinec, 2023) with the *ordbetareg* package in R with R studio. The OBR models consider values that lie at the bounds of 0 and 1, which the traditional beta regression models do not. As a gaussian prior is inbuilt for the coefficients in the package we set the mean at 0 and the standard deviation at 2. We post-hoc defined the contrast for the independent variables of interest which were the AOI and the strategy, through the *emmeans* package in R. Finally, we calculated the Bayes Factor for the effect through the Savage Dickey Density Ratio.

The first analysis was to verify if we were able to observe LAN to L1 for the three types of probes: positive, lure and new probes. The former two had been implemented in a previous study (Bhanap et al., 2025) and reliably had produced LAN. Here, we additionally checked if we observe the same pattern of results also for new probe trials. The analysis was conducted separately for the WM retrieval and LTM retrieval phase. For both retrieval phases, we analysed the eye-tracking data within the time window starting from the onset of the first probe until the participants made their response. In a similar previous study, we reliably observed LAN towards the location of L1 throughout different time windows of the retrieval phase, that is, first and second probe interval, the interval between probe presentations, and the time until participants gave their response (Bhanap et al., 2025). Therefore, we pooled the data across all the time windows of this study in the subsequent analysis. The analyses separated by time window are included in the supplementary materials. We included AOI and probe type as fixed effects in the model. For this and all subsequent models we included the full random effects structure, justified by the design (including random slopes for the fixed effects as well as subjects as a random intercept). The second analysis pertained to investigate if we observe LAN for the different strategies. For this analysis, we pooled the data across the probe types and ran a model with strategy and AOI as the fixed effects as well as including their interaction.

Third, we compared the amount of LAN observed in each strategy to the other strategy conditions. Thus, we compared the fixation proportion to L1 throughout the retrieval phase by running a model including strategy as a fixed effect and for which fixation proportion to L1 was the dependent variable.

Finally, we wanted to test if any strategy had an impact on the functionality of LAN. For that we analyzed fixation proportions to L1 for trials in which participants gave a correct response versus an incorrect response for each strategy, by entering the main effects and interaction of correctness of responses and strategy into our OBR. We ran this analysis for fixations during the time frame of the WM test, LTM test and for the retention interval. We defined the AOIs in the same way as during the retrieval phase (e.g., L1 being defined as the location of the probed word pair) – although in case of the retention interval, participants would not have known which of the word pairs would be tested and what L1 would be. This coding scheme, however, allowed us to test whether the looking behavior in the retention interval influences memory performance in the retrieval phase.

For each model, the samples were generated with 25000 iterations with 2000 of them allocated as warmup samples and are generated through 4 Markov Chains. Before calculating the Bayes Factors, we verified if the models converged by checking that the R-hat statistic was <1.05.

### Results

#### Behavioral Results

**Figure 2:**
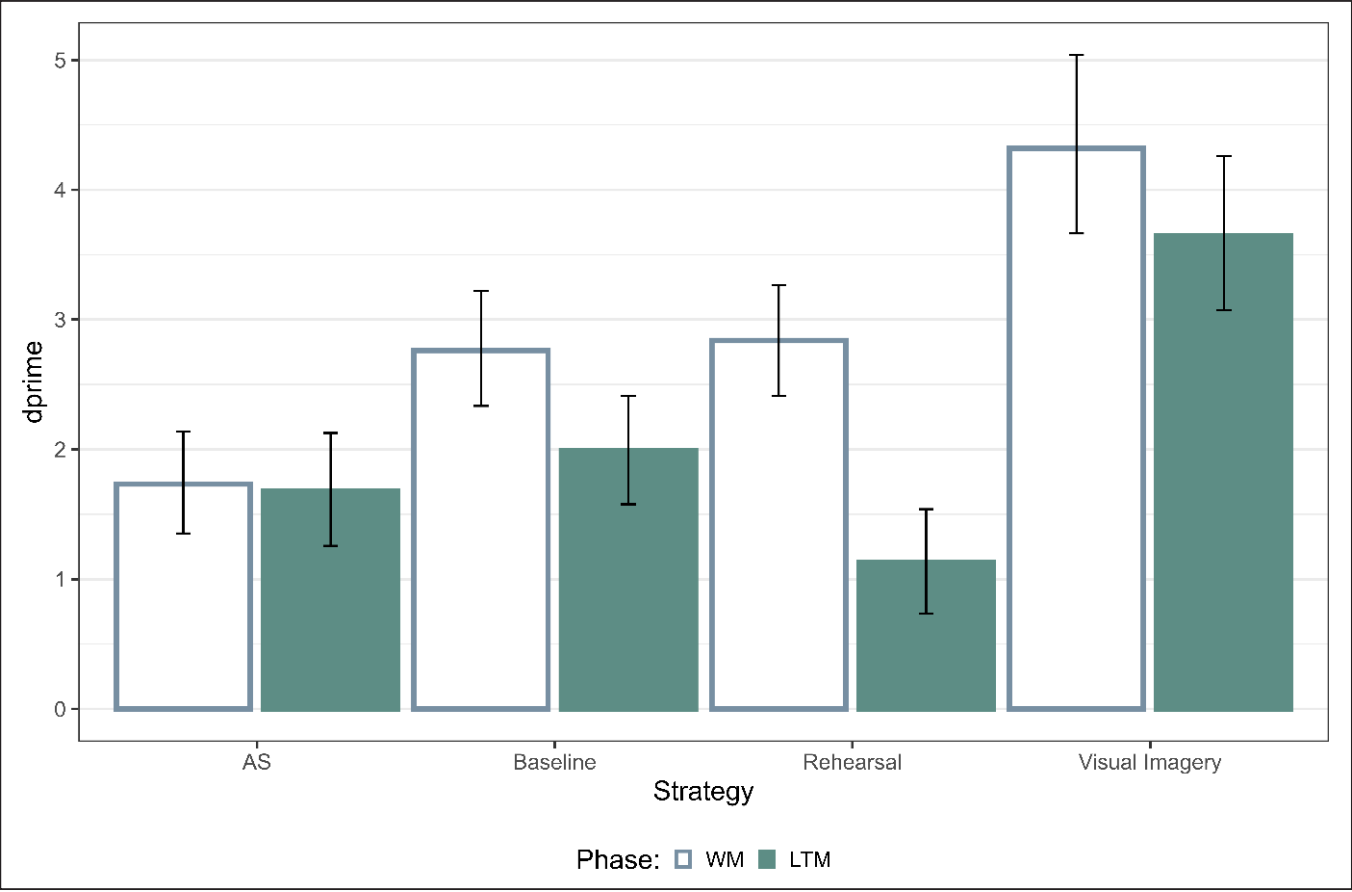
Memory performance for the WM and LTM Test. The error bars represent 95% highest posterior density intervals (HPD). Note: AS refers to articulatory suppression.

First, we were interested in the accuracy data across different strategy conditions by calculating dprime from the posterior estimates of the GLMs (Figure 2). For the WM test, memory performance was the highest for visual imagery, followed by baseline and rehearsal and lowest for articulatory suppression (Table 1). For the LTM test, memory performance was the highest for visual imagery, followed by baseline, articulatory suppression and lowest for rehearsal (Table 1). This together replicates previous findings, showing that rehearsal and visual imagery result in differential effects on WM and LTM.

**Table 1:** Bayes Factors (BF_10_) for pairwise comparisons on the main effect of strategies on the measure of dprime for both the WM and LTM Test.


STRATEGY	BASELINE		REHEARSAL		VISUAL IMAGERY	
		
	WM TEST	LTM TEST	WM TEST	LTM TEST	WM TEST	LTM TEST

AS	1.7 × 10^2^	0.30	5.7 × 10^2^	1.44	4.5 × 10^7^	7.5 × 10^4^

Baseline			0.17	80.51	8.9 × 10^2^	6.3 × 10^3^

Rehearsal					1.6 × 10^3^	4.4 × 10^6^


Note: AS refers to articulatory suppression.

Pertaining to the question of whether participants complied to our instructions, we took the following measures: first, we only included trials where participants indicated compliance to the strategies (94% of all trials). Furthermore, we inspected the LTM data and were able to observe a strong benefit of visual imagery on LTM performance and therefore, we could be certain that participants engaged in visual imagery (see Bartsch et al., 2018 for a similar approach). Finally, independent research assistants inspected the auditory files for rehearsal and articulatory suppression and ensured that participants engaged in the respective strategy for all the trials.

#### Eye Movement Results

##### LAN during the WM and LTM test

**Figure 3:**
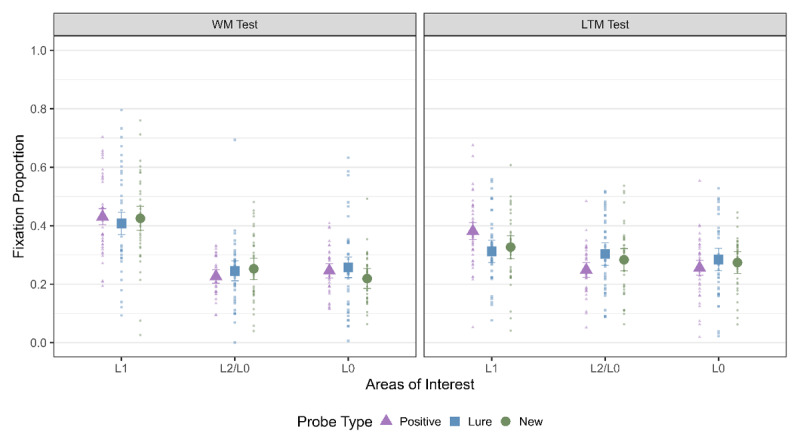
Fixation Proportion to the three AOIs across strategies for both WM and LTM Test. The error bars indicate 95% within subject confidence intervals.

Here, our first step was to investigate if we observe LAN to L1 during the WM test and thereby replicate the results from Bhanap and colleagues (2024) and additionally test whether we also observe LAN in the LTM test (Figure 3). For the WM test, we observed strong evidence in favor of a main effect of AOI, which indicated that LAN was observed to L1 for all three probe types. For the LTM test, we observed evidence for a main effect of AOI for positive trials, evidence *against* it for lure trials and inconclusive evidence in case of new trials. Thus, we observed a clear effect of LAN to L1 during the WM test but for the LTM test, the results were more ambiguous (Table 2). The primary objective of the LTM test was to ensure that participants engaged in visual imagery, as previous research suggests that visual imagery enhances LTM retrieval but has limited impact on WM performance. In the LTM test, we retested the word pairs that were previously included in the WM test. This retesting may have strengthened the representations of these word pairs, as demonstrated by Roediger and Karpicke (2006), potentially contributing to the ambiguous results for LAN, consistent with Scholz et al. (2011). Alternatively, previous research also suggests that untested word pairs on each trial could have experienced retrieval-induced forgetting (e.g., Anderson et al., 2000), where competing, untested word pairs may show poorer LTM performance compared to the tested word pairs.

**Table 2:** Bayes Factor (BF_10_) for a main effect of AOI across probe types for both WM and LTM Test. The AOIs were contrast coded as L1(2) and L2/L0 (–1).


PROBE TYPE	WM TEST	LTM TEST

Positive	1.08 × 10^5^	2.5 × 10^2^

Lure	7.04 × 10^3^	0.08

New	2.75 × 10^4^	0.52


Furthermore, in the LTM test, each location was overloaded with 48 word-pairs, reducing the distinctiveness of location information compared to the WM test, where each word pair was uniquely associated with a specific location on every trial. Future studies should account for these factors, including the testing effect and retrieval-induced forgetting, to better isolate and clarify the relationship between LTM and LAN.

**Figure 4:**
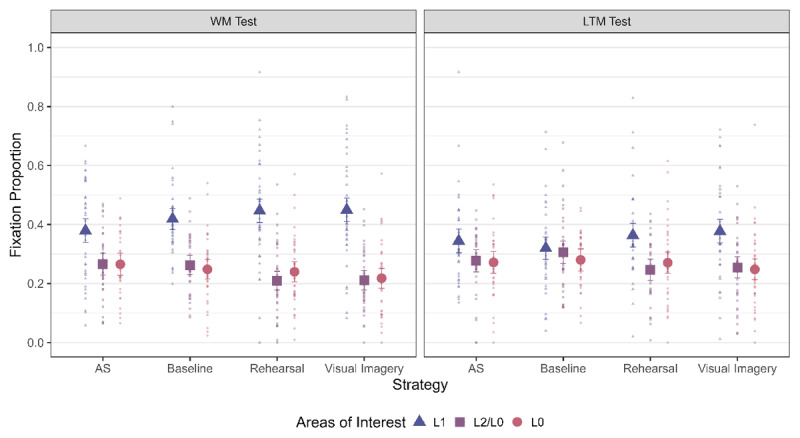
Fixation Proportion towards the three areas of interest across strategies and for the two phases. The error bars indicate 95% within subject confidence intervals.

The next step was to check if we observed LAN across all strategy conditions for both the WM and LTM test (Figure 4). This was the critical test to distinguish between the Imagery LAN and Memory Strength LAN hypothesis. As can be seen from Table 3, we observed LAN to L1 for all strategy conditions in the WM test – which speaks against the Imagery LAN hypothesis which predicted LAN specifically for visual imagery but not for rehearsal. Furthermore, it indicates that location information is indeed reactivated during rehearsal – contrary to what is predicted by Baddeley (1996).

**Table 3:** Bayes Factor (BF_10_) for a main effect of AOI across strategies for both WM and LTM Test. The AOIs were contrast coded as L1 (2) and L2/L0 (–1).


STRATEGY	WM TEST	LTM TEST

AS	4.23	0.52

Baseline	1.42 × 10^3^	0.05

Rehearsal	2.93 × 10^3^	7.62

Visual Imagery	8.20 × 10^4^	12.95


When it comes to the LTM test, the picture is less clear. We observed moderate evidence in favor of the main effect of AOI for Rehearsal and Visual Imagery, inconclusive evidence for AS and evidence against a difference in Baseline (see Table 3). Thus, there is a clear LAN effect to L1 during the WM test for all strategy conditions and for most strategy conditions in the LTM test. This indicates that location information is reactivated during retrieval, both from WM as well as after a delay.

##### Differences in LAN across Strategies

The next question was whether the amount of LAN to L1 differed across the different strategy conditions for the WM retrieval phase. As can be seen from Figure 4 and Table 4, we observed evidence *against* a difference for all the comparisons. Thus, we overall observed no differences in the amount of LAN observed across the different strategy conditions – again speaking against the Imagery LAN hypothesis.

**Table 4:** Bayes Factor (BF_10_) for the pairwise comparisons between strategies for fixation proportion to L1 for both the WM and LTM Test.


STRATEGY	BASELINE		REHEARSAL		VISUAL IMAGERY	
		
	WM TEST	LTM TEST	WM TEST	LTM TEST	WM TEST	LTM TEST

AS	0.13	0.06	0.09	0.05	0.14	0.05

Baseline			0.04	0.16	0.04	0.11

Rehearsal					0.05	0.04


As reported by Bhanap and colleagues (2024), the amount of LAN that is observed throughout a retrieval phase can change across the timeline. Accordingly, we formalized this in an analysis to see if Visual Imagery lead to more LAN during these time points. We ran the same ordered beta regression models as before, but we restricted the data to the probe interval till the end of the second probe. We did not see any credible difference in the amount of LAN observed across the strategies even for this duration. Thus, the amount of LAN observed does not differ across strategies. The plots and the analysis can be found in the supplementary materials.

Next, we looked at whether the amount of LAN observed in the LTM test differed across the strategies. The analysis was run throughout the retrieval phase (comprising first and second probe, inter-probe-interval and response phase). We observed evidence *against* a main effect of strategy for all comparisons (Table 4). Thus, there was no difference in the amount of LAN that was observed across strategies in the LTM retrieval phase. Additionally, we looked at whether the fixation proportions to L1 differed between the strategies during the inter-word interval and second probe. However, there was evidence against, all the main effects. Thus, the amount of LAN does not differ during LTM retrieval for information that was encoded under different strategy instructions.

##### Eye Movement Functionality

**Figure 5:**
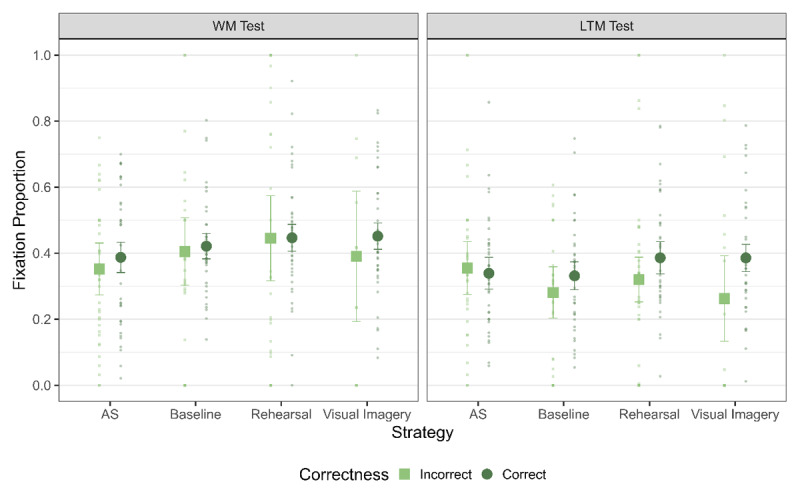
Fixation Proportion to L1 across the strategies for trials with correct and incorrect responses for the WM Test. The error bars indicate 95% of within subject confidence intervals.

Finally, we ran the analysis to investigate the functionality of LAN across strategies – that is, whether the amount of LAN observed in trials was affected by the correctness of the trial differentially across strategies (Figure 5). For the WM test, we observed evidence against a difference between fixation proportions for correct and incorrect trials (main effect of correctness: BF_10_ = 0.30). Additionally, there was no differential effect of correctness across the strategy conditions (interaction of correctness and strategy: BF_10_ = 0.07). Thus, the amount of LAN was not functionally related in any of the strategies to memory performance in the WM test and between different strategy conditions (see Table 5).

**Table 5:** Bayes Factors (BF_10_) for the main effect of correctness for fixation proportion to L1 for WM test, LTM test and Retention Interval.


STRATEGY	WM TEST	LTM TEST	RETENTION INTERVAL

AS	0.06	0.05	0.05

Baseline	0.06	0.07	0.07

Rehearsal	0.05	0.08	0.05

Visual Imagery	0.11	0.10	0.09


For the LTM test, we found inconclusive evidence for a main effect of correctness (BF_10_ = 0.48) and evidence against a main effect of strategy (BF_10_ = 0.05) as well as for the interaction between correctness and strategy (BF_10_ = 0.29). Similarly, for retention interval, we also obtained evidence against a difference for Baseline and inconclusive evidence for AS, Rehearsal and Visual Imagery (Table 5, see supplementary materials for Figure). Thus, for neither the WM, nor the LTM test or the retention interval, did we find evidence for a link between correctness of response and LAN behavior.

### Discussion

The first goal of Experiment 1 was to investigate a prediction of the multicomponent model of WM, specifically, that location information is reactivated for visual imagery but not for rehearsal. We employed LAN as a tool to assess the activation of location information. Within the model, Baddeley (1996) proposed that rehearsal engages the phonological loop activating the item information but not the spatial location, whereas visual imagery engages the visuospatial sketchpad and therefore activates the item and spatial information. Here, we observed that location information is activated not only for visual imagery but also for rehearsal. The results indicate that the separation of mechanisms between rehearsal and visual imagery is not as strict as initially proposed and both lead to reactivation of item and location information.

Our second goal was to determine what drives the strength of the LAN effect, whether it is the engagement in visual imagery or the strengthening of WM traces. We considered two hypotheses: the Imagery LAN hypothesis and the Memory Strength LAN hypothesis. First, the Imagery LAN hypothesis predicted LAN to be observed when people engage in visual imagery. Our results of Experiment 1 speak against this as LAN was not only observed when participants engaged in visual imagery but also when they were instructed to engage in rehearsal.

The present study is the first to compare instructions of visual imagery to other strategy instructions on the effect of LAN. Our results show how critical such comparison conditions are for understanding the phenomenon. Therefore, the strict notion – derived from theories like the Enactive Theory (Thomas, 2009) and the Visual Buffer Model (Kosslyn et al. 2006) – that LAN is observed in the service of building and inspecting mental images and in the absence of them, LAN will not be observed, cannot hold. The results align with Ferreira and colleagues (2008), who suggest that item and location information are stored as an integrated representation, such that retrieving one naturally leads to the retrieval of the other and this leads to eye movements to associated spatial locations. Indeed, our findings show that during rehearsal, when item information is recalled, location information is also retrieved, which is evidenced by the presence of LAN. Thus, the results cannot be explained by the Imagery LAN hypothesis.

Second, we considered the Memory Strength LAN hypothesis. In contrast to previous studies on WM performance and visual imagery (e.g., Bartsch 2018, 2019; but see Bartsch et al. 2024 for similar results), we observed the highest immediate memory performance in the WM test for visual imagery instructions. According to the Memory Strength LAN hypothesis, as the visual imagery instruction seemed to have increased the strength of WM traces, we should also see an increase in the amount of LAN we observed, compared to the other instructions. However, this is not what we observed. Instead, we found similar amounts of LAN across all the strategy conditions. Thus, both the Memory Strength LAN and Imagery LAN hypothesis cannot explain the results of the study.

A way to salvage the Memory Strength LAN hypothesis is to assume that visual imagery increases memory strength of LTM traces, which in turn enhances immediate memory performance, yet that WM traces were unaffected by visual imagery. Previous studies (Bartsch et al., 2024) support this notion, showing that visual imagery can enhance LTM trace strength, which might subsequently be drawn upon in WM tasks. If WM performance in the visual imagery condition of Experiment 1 was primarily driven by these strengthened LTM traces, it would explain the high performance without a corresponding increase in LAN, as LTM trace strength might not directly influence LAN (see Vieira et al. 2023).

If our high WM performance in visual imagery is indeed due to the influence of LTM traces, this would explain why LAN does not show a corresponding increase, highlighting the need to further investigate the relationship between LTM trace strengthening and WM performance in visual imagery. Another possibility, although less plausible given the lack of supporting evidence in previous studies, is that visual imagery in our paradigm might have enhanced the strength of WM traces, thereby improving immediate memory performance but not enhance LAN.

To address this unresolved issue, Experiment 2 was sought out to test the hypothesis that the high WM performance in visual imagery is driven by the strengthening of LTM traces rather than WM trace strength. By investigating whether LTM trace strengthening is the key factor behind the observed performance, we aim to clarify the relationship between visual imagery, WM performance, and LAN.

## Experiment 2

In Experiment 2, we aimed to test if the WM benefit for visual imagery in Experiment 1 was indeed driven by strengthening of LTM traces and whether we can salvage the Memory Strength LAN hypothesis, by reducing the contribution of LTM traces in the WM test. To this end, we will adopt the experimental manipulation of proactive interference from Bartsch and Oberauer (2023). As stated before, proactive interference affects retrieval from LTM. Therefore, for Experiment 2, we changed the procedure of Experiment 1, in the following way: First, we ran two sessions of the experiment for each participant. One session introduced proactive interference (PI Block), in which word pairs were formed from a small pool of nine words. The other session realized the no proactive interference session (No PI Block) similar to Experiment 1. The order of the sessions was counterbalanced between participants.

If the increase in immediate memory performance under visual imagery compared to the other strategies is driven by the strengthening of LTM traces, we will observe a decrease in WM performance for this condition in the PI Block. In that case, we expect to observe the same amount of LAN for visual imagery as compared to other strategy conditions. For the No PI block, we expect to replicate the results from Experiment 1 and observe higher WM performance and the same level of LAN for visual imagery compared to the other strategies.

An additional consideration for the observed LAN results is the potential impact of task demands during the PI block. The constant repetition of words within this block may lead to increased cognitive load as participants repeatedly encounter the same words. Under such circumstances, participants might rely more on LAN to resolve the task at hand. This increased reliance could lead to higher LAN during the PI block compared to the NoPI block. Previous research on LAN supports this perspective, suggesting that retrieving difficult words imposes a higher memory load. According to Kumcu and Thompson (2018), such conditions may enhance the appeal of environmental support for memory retrieval. This implies that LAN could serve as a compensatory mechanism under heightened task demands. To further investigate this, we will compare LAN between the PI and NoPI blocks across strategies. This analysis will help determine whether the observed LAN effects exhibit a general decrease under varying task demands or not.

### Methods

#### Participants

We collected data of 39 participants. The inclusion criteria from Experiment 1 were also applied for this experiment. As preregistered, we first collected data of 30 participants and continued data collection till the Bayes Factor for the accuracy analysis was over 3 in support or against the comparison between PI and the No PI block for visual imagery. Additionally, we replaced 2 participants whose mean performance was below chance level and excluded 10 participants who did not meet the dwell criterion for the eye tracking data. Data from 2 participants had to be excluded due to loss of eye tracking data. Thus, our final sample consisted of 26 participants (M_age_ = 24.8, SD_age_ = 5.4, 21 female).

#### Materials and Procedure

The procedure for Experiment 2 was similar to Experiment 1 except for the following changes. Each participant took part in two sessions: a PI and a No PI session. In the PI session, participants were presented with word-pairs formed each trial from a small pool of 9 words. Thus, in every trial six words were randomly chosen from this pool to form three word-pairs. In the NoPI session, we used a large pool of words, with new words forming three word-pairs on every trial. Each block contained 48 trials and participants had a 20-minute break in between the two sessions. The order of the blocks was counterbalanced across the participants. As the PI session repeated the same words across many trials and conditions, we could not have a meaningful LTM test, thus, we eliminated the distractor filled delay and LTM test.

The final change in the design pertains to replacing the trials with “new” probes in the retrieval phase with “old-trial lures”. An old-trial lure was a word used in a previous trial, specifically the n-1 trial. Bartsch and Oberauer (2023) reported that the probability of choosing an old-trial lure increases in a PI block as compared to a No PI block indicating that the small pool of words is affecting retrieval from LTM. By contrast, the probability of choosing new words does not change between PI or No PI blocks. Therefore, to increase the impairment caused by PI on the LTM traces, we decided to include old-trial lures. For the PI block, this lure had been associated with the probe word in the previous trial. For the No PI block, the lure was randomly chosen from the six words from the previous trial. For clarity, we refer to the lures from within the same trial as within-trial lure.

### Results

#### Behavioral Results

**Figure 6:**
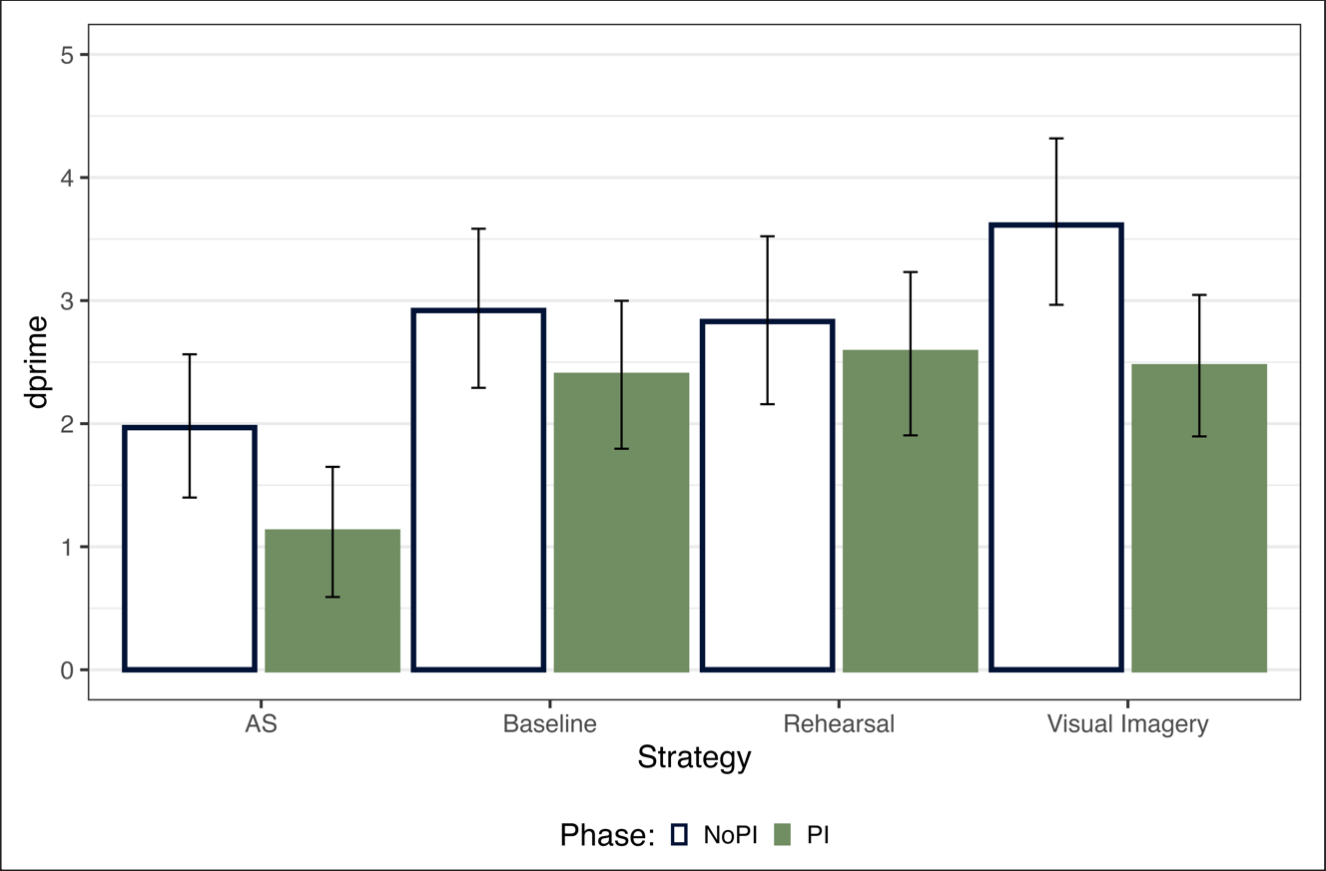
dprime across the PI and No PI Block. The error bars indicate 95% highest posterior density intervals.

Like in Experiment 1, we estimated dprime across the two PI sessions and strategies from the Bayesian GLM based on the accuracy results (Figure 6). We compared each strategy condition and the effect of PI through pairwise comparison using the *emmeans* package.

Our results showed that in the No PI session, memory performance was highest for visual imagery and lowest for articulatory suppression (Table 6). Thus, we replicated the findings from Experiment 1. The evidence for a difference between Baseline and Rehearsal with Visual Imagery is inconclusive. In the PI session, we saw no difference between visual imagery, baseline and rehearsal and the lowest memory performance for articulatory suppression. Thus, the WM performance benefit for visual imagery was absent in the PI session, replicating results from Bartsch and colleagues (2024). Additionally, we observed a main effect of PI session for visual imagery (BF_10_ = 17.26), indicating that the memory performance reduced from NoPI to PI session.

**Table 6:** Bayes Factor (BF_10_) for a main effect of strategy on the measure of dprime across the two PI sessions.


STRATEGY	BASELINE		REHEARSAL		VISUAL IMAGERY	
		
	PI	NO PI	PI	NO PI	PI	NO PI

AS	91.43	4.02	3.1 × 10^2^	2.00	4.3 × 10^2^	3.3 × 10^2^

Baseline			0.22	0.21	0.16	0.90

Rehearsal					0.19	1.02


#### Eye Movement Results

##### LAN during the WM test

We used the same Areas of Interest (AOI) and calculated the fixation proportions in the same manner as for Experiment 1.

**Figure 7:**
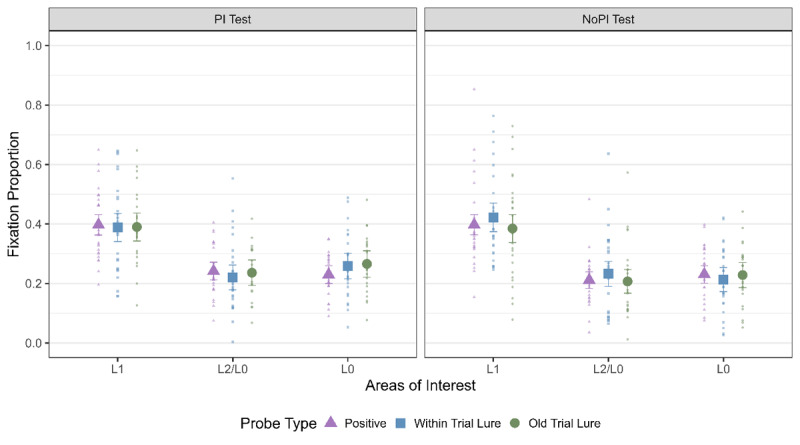
Fixation Proportion to all three areas of interest for all the three probe types for both the No PI and PI session. The error bars indicate 95% within subject confidence intervals.

We first verified if we observed LAN to L1 as in Experiment 1. As can be seen from Table 7 and Figure 7, we observed a main effect of AOI for all probe types and for both the PI sessions (Table 7). Thus, we observed LAN to L1 as in Experiment 1 and as observed by Bhanap and colleagues (2024).

**Table 7:** Bayes Factor (BF_10_) for a main effect of AOI across probe types for both PI and NoPI Test. The AOIs were contrast coded as L1(2) and L2/L0 (–1).


PROBE TYPE	PI TEST	NOPI TEST

Positive	98.17	3.01 × 10^2^

Within Trial Lure	49.81	6.37 × 10^2^

Old Trial Lure	6.15	83.87


**Figure 8:**
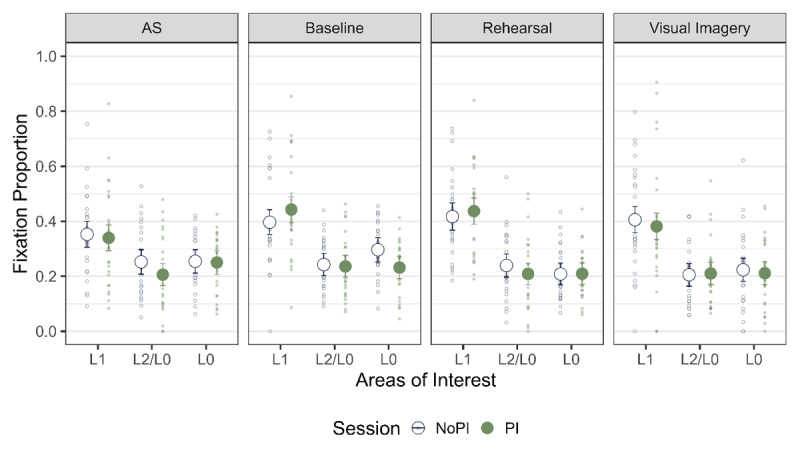
Fixation Proportion across the two phases and across all the strategies. The error bars indicate 95% within subject confidence intervals.

Next, we tested if we observed LAN to L1 for all the strategy conditions across the two PI sessions (Figure 8). We pooled the data over the three probe types and compared the fixation proportion to L1 with that of L2 and L0 with the same contrast coding as mentioned before. We observed the main effect of AOI for rehearsal and visual imagery for both the PI sessions and for baseline in the NoPI session (Table 8). We observed inconclusive evidence for AS for both the PI sessions and for baseline in the PI session. Thus, LAN was observed across most strategy conditions.

**Table 8:** Bayes Factor (BF_10_) for a main effect of AOI across strategies for both PI and NoPI Test. The AOIs were contrast coded as L1 (2) and L2/L0 (–1).


STRATEGY	PI TEST	NOPI TEST

AS	0.4	0.52

Baseline	2.87	1.3 × 10^3^

Rehearsal	3.6 × 10^2^	4.3 × 10^3^

Visual Imagery	80.61	58.27


##### Differences in LAN across Strategies

Next, we compared the amount of LAN to L1 across different strategies (Table 9). We found evidence against a main effect of strategy on fixation proportions to L1 for comparisons between baseline, rehearsal and visual imagery in both the PI sessions. When zooming in on the No PI session, we observed inconclusive evidence for a difference between baseline and AS as well as rehearsal with AS. In the PI session, we found evidence against a difference between AS and other strategy conditions. There was inconclusive evidence for the comparison of AS to baseline. Thus, overall, we did not observe difference in LAN between strategies except for some comparisons in the NoPI condition, where we observed inconclusive evidence.

**Table 9:** Bayes Factor (BF_10_) for a main effect of strategies for fixation proportion to L1 for both PI and No PI session.


STRATEGY	BASELINE		REHEARSAL		VISUAL IMAGERY	
		
	NO PI	PI	NO PI	PI	NO PI	PI

AS	0.38	0.06	1.06	0.15	0.06	0.06

Baseline			0.04	0.05	0.12	0.06

Rehearsal					0.21	0.09


As we looked at whether the amount of LAN differed during the time duration from beginning of inter-probe-interval till the end of the second probe in Experiment 1, we ran a similar analysis for Experiment 2. We observed the same result as Experiment 1. That is, we did not observe any differences in the amount of LAN between Baseline, Rehearsal and Visual Imagery. The plot and analysis can be found in the supplementary material.

Additionally, we investigated whether the effect of LAN to L1 differed between the PI and No PI session across all the strategy conditions. We found evidence against such a difference for all the strategies. Thus, there was no effect of PI on LAN, meaning that even though the contribution from LTM on immediate performance was impaired via the introduction of PI, LAN was not affected by it (Table 10). Our findings indicate that LAN is not affected by the higher task demands of the PI block. One possible explanation for this is that the increased task demands in the PI block influence only the LTM traces rather than WM traces. In this scenario, the individual WM traces created for word pairs on each trial may remain consistent in their immediate test demands, resulting in similar LAN for both the PI and NoPI blocks. The observed stability in LAN across conditions states that WM traces are not affected by these additional task demands; thus, the task can be performed without additional reliance on LAN.

**Table 10:** Bayes Factor (BF_10_) for a main effect of PI Session for fixation proportion to L1 across all the strategies.


STRATEGY	BF_10_

AS	0.04

Baseline	0.05

Rehearsal	0.03

Visual Imagery	0.03


##### Eye Movement Functionality

**Figure 9:**
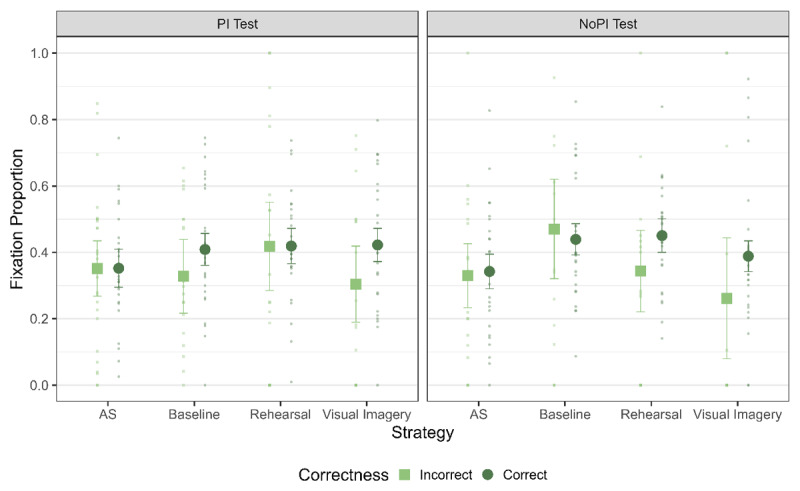
Fixation Proportion towards L1 across the two phases and correctness on the trial. The error bars indicate 95% within subject confidence intervals.

The final step of the analysis was to test if the amount of LAN observed during the WM retrieval phase differed on trials in which participants gave a correct versus an incorrect response (see Figure 9). We ran a model with correctness as a predictor for the fixation proportion to L1 across all the strategies and the PI sessions. We find evidence against a main effect of correctness of response across all the strategies and PI sessions (Table 11). Additionally, we also analyzed if there is a relation between proportion of fixations in the retention interval and the eventual memory performance in the WM task. Again, we observed no evidence in favor of a difference in LAN for correct and incorrect trials (Table 11).

**Table 11:** Bayes Factor (BF_10_) for a main effect of correctness of response for fixation proportion to L1 for PI and No PI sessions and for their respective Retention Intervals.


STRATEGY	WM TEST		RETENTION INTERVAL	
	
	PI	NO PI	PI	NO PI

AS	0.04	0.05	0.05	0.69

Baseline	0.15	0.05	0.04	0.17

Rehearsal	0.06	0.20	0.05	0.05

Visual Imagery	0.10	0.18	0.05	0.09


### Discussion

In Experiment 2, we replicated the WM benefit for visual imagery reported in Experiment 1 for the No PI session. Critically, the introduction of PI fully eliminated this benefit – replicating the findings from Bartsch and colleagues (2024). Both the present and previous results therefore speak towards the WM benefit for visual imagery being driven by strengthening of LTM traces.

Furthermore, we observed the same level of immediate memory performance for PI and No PI sessions under instructions of rehearsal and baseline, indicating that the performance in these two conditions was not driven by LTM traces. Finally, we saw that WM performance for AS was negatively impacted by PI. Similar results were found in a study by Atkins and colleagues (2011), in which the introduction of AS in a PI condition further decreased memory performance of participants. They suggested that AS interferes with rehearsal processes and reduces the distinctiveness of the current memoranda by decreasing the signal to noise ratio, which further impairs the performance in the PI condition. We can think of another explanation which is that memory performance in the AS condition (without PI) could be driven by LTM traces as phonological traces in WM are blocked. These LTM traces could be rapidly formed during the WM task (as proposed by Cowan, 2019) and should be unaffected by AS. Yet, in the PI session, these LTM traces are negatively affected by an additional source of interference (proactive), thus leading to an additional decrease in memory performance.

Concerning the eye movement data, we observed LAN to L1 for both rehearsal and visual imagery. This is a strong indication that location information is indeed reactivated for both these strategies, replicating the results from Experiment 1.

Although PI clearly affected WM performance in the visual imagery condition, the amount of LAN was unaffected. In other words, the decrease in strength of LTM traces in the PI condition did not lead to any changes in LAN, aligning with the findings from Vieira and colleagues (2023). Our results point more towards an explanation, that visual imagery drives the modulation in memory strength and LAN via two separate mechanisms. We discuss the implications of this in more detail in the General Discussion.

## General Discussion

The aim of the present study was two-fold. First, we set out to test the theoretical notion whether rehearsal and visual imagery are driven by two separate mechanisms, whereby location information is reactivated only for visual imagery – as predicted by common memory models like the multi-component model (Baddeley 2000). To this end, we built on previous research showing that eye movements can serve as a tool for revealing the activation of location information. Our results show that both rehearsal and visual imagery reactivate location information – as evident in LAN to L1 for both rehearsal and visual imagery conditions. Thus, our current results do not support the idea that location information is reactivated for visual imagery but not for rehearsal.

Second, we aimed to further investigate what drives LAN in a WM task by testing two hypotheses about the mechanisms underlying eye movements during visual imagery: the *Visual Imagery LAN* hypothesis and the *Memory Strength LAN* hypothesis.

The *Visual Imagery LAN* hypothesis suggests that the LAN effect arises from the creation and inspection of mental images. If this were true, we would expect stronger LAN effects for visual imagery and little to no LAN in other conditions. However, our results showed similar levels of LAN across visual imagery, rehearsal, and baseline conditions, contradicting this hypothesis. The *Memory Strength LAN* hypothesis proposes that LAN is not tied to a specific strategy but instead reflects the strength of WM traces. According to this view, LAN should correlate with WM performance, meaning stronger LAN effects should be observed for visual imagery, which led to the highest WM performance. However, our findings also did not support this prediction.

Building on this, Experiment 2 aimed to determine whether the benefits of visual imagery in WM are driven by strengthening of LTM traces as previously proposed. The reduced WM benefit from visual imagery following the introduction of PI suggests that memory performance for visual imagery is indeed driven by strengthening of LTM traces. The behavioral findings replicate those of Bartsch and colleagues (2024).

Lastly, pertaining to our second goal, we showed that both our original hypotheses on the mechanisms underlying eye movements in WM cannot explain our results. Instead, we argue that our results regarding LAN are best explained by selection processes in the FoA which feed into the eye movement system. In the following we discuss the possible mechanisms that may drive LAN for visual imagery in WM.

### Implications for the relation between Visual Imagery and LAN

Previous research on LAN has primarily – but not exclusively (see Kumcu & Thompson, 2018; Scholz et al., 2016) – focused on eye movements during visual imagery, consistently reporting the presence of LAN during both the creation and inspection of mental images (Maratelli & Mast, 2013; Umar et al., 2021). These findings have been interpreted as evidence that LAN is specifically tied to the visual processing required for mental imagery. According to this view, LAN should be exclusive to visual imagery. However, this interpretation is limited by the fact that prior studies only examined visual imagery instructions, without comparing LAN to a baseline or other strategy conditions. In our study, we observed LAN not only during visual imagery but also under verbal rehearsal, articulatory suppression, and even in a baseline condition where participants were free to use any strategy or none at all. These findings strongly suggest that LAN is not tied exclusively to visual imagery, prompting the need for alternative explanations.

One possible explanation is the mental simulation account, which posits that mental simulation is a general cognitive mechanism, not limited to visual imagery (Kosslyn et al. 2006). This implies that participants engaged in some form of mental simulation, even under rehearsal and baseline conditions, leading to similar levels of LAN across these conditions. However, two aspects of our results challenge this explanation. First, if mental simulations were equally employed across all conditions, we would expect similar performance outcomes. However, only the visual imagery instruction led to higher immediate memory (in Exp. 1 and in the No PI condition of Exp 2) and delayed memory performance (Exp. 1), suggesting that elaborative visual imagery was more prominent in this condition. Second, the idea of widespread mental simulation contradicts the known effects of AS, which impairs verbal rehearsal but does not suppress mental simulation. If participants were engaging in active mental simulation across conditions, AS should not uniformly impair LAN, yet it does, indicating that mental simulation is not the sole factor driving LAN.

Another explanation is that participants might have used spatial cues during the retention interval to engage in spatial rehearsal alongside verbal rehearsal. For example, during rehearsal or baseline conditions, participants could have rehearsed both item and location information by using spatial cues as an aid. This idea is supported by findings from Tremblay and colleagues (2006), who observed that participants made eye movements to the location of dots still present on screen during a task, reflecting spatial rehearsal. While in their study, the location of the dots was relevant, in our study the location was irrelevant. It could be that the location of the information is incidentally retrieved and recalled even when participants are asked to engage in a predominantly verbal strategy. This may lead to the recall of not only the verbal information but also the spatial information. Thus, leading to the reactivation of both the item and location information. For instance, Awh and Jonides (2001) propose that spatial attention is employed to rehearse and maintain information actively in WM. Thus, eye movements are used to maintain a representation of space. These proposals further suggest that eye movements made during rehearsal are integral to the rehearsal process, enhancing memory performance for the items that are revisited or rehearsed. However, it is possible that the location information is sequentially brought to the FoA, leading to eye movements towards those locations.

Additionally, we used commonly known instructions to guide participants in employing different strategies. However, from a theoretical perspective, it is not immediately clear that individuals will naturally adopt a spatial rehearsal strategy in a paradigm in which location is entirely irrelevant for solving the task. Our findings demonstrate that both strategies facilitate the retrieval of location information, as evidenced by eye movements, which served as a process measure for the reactivation of spatial memory. Future research on strategy use should take greater care in manipulating and assessing strategy application. In particular, studies could have better control for the extent to which individuals rely on spatial information when using these strategies, especially if the goal is to clarify their underlying mechanisms.

Additionally, a range of previous studies propose incidental binding of verbal and spatial information. For instance, Guérard and colleagues (2009, 2013) proposed that verbal and spatial information are incidentally bound to each other. One of them acts as the primary feature which can be used for the retrieval of the linked feature. Their results show that serial presentation of information at the time of encoding leads to a situation where location information is the primary feature and can act as the retrieval cue for the verbal information. By contrast, sequential presentation of information leads to a situation where the verbal information acts the primary feature. Nonetheless they are both linked to each other. Thus, in this context the presence of LAN for rehearsal and visual imagery seems a plausible outcome, considering the retrieval of verbal information during rehearsal can lead to the retrieval of spatial information. While some other studies propose that visual and verbal information may have separate stores (i.e. the visuospatial sketchpad and the phonological loop), they are not completely distinct. They rather share similar underlying processes for retention of verbal and visual information (Meiser & Klauer,1999; Marsh et al., 2024). The findings can still be explained within the context of the multicomponent model by considering the role of the episodic buffer (Baddeley, 2000). The episodic buffer integrates information from multiple sources and stores it in a multidimensional code, capable of handling visual, verbal, and spatial information. For articulatory rehearsal, spatial information might be processed in the episodic buffer alongside item activation in the phonological loop, leading to the observed LAN.

Given the similar levels of LAN for both rehearsal and visual imagery, it is plausible that visual imagery also relies on a shared underlying mechanism involving the episodic buffer. Thus, the episodic buffer‘s role should be accounted for when evaluating different memory strategies. However, in its current state, the multicomponent model of memory does not propose a mechanism that can lead to LAN. An alternative explanation to consider could be the shared priority map framework.

According to this framework, LAN is not tied to visual processing alone but arises from the retrieval of item-location bindings into the FoA (focus of attention, which is a selection mechanism for WM), which leads to a shift of attention toward associated locations (Hedge et al., 2015; Theeuwes et al., 2009; Bhanap et al., 2025) This mechanism, which manifests as LAN, is consistent with the integrated representation idea proposed by Ferreira and colleagues (2008), where the retrieval of one element (e.g., item information) naturally leads to the retrieval of another (e.g., spatial location). This interpretation also aligns with the gaze reinstatement hypothesis, which suggests that eye movements during retrieval reflect the reinstatement of scan paths embedded in memory representations (Wynn et al., 2022; Johansson et al., 2022). Similarly, it accounts for the external memory account of Richardson and Spivey (2000), which posits that when people encode item information, they store spatial tags along with it and at the time of retrieval of the item information, these spatial tags (location information) are retrieved, thus leading to LAN. The shared priority map framework thus provides a coherent explanation for LAN that extends beyond visual imagery, applying equally to baseline and rehearsal conditions. Unlike the alternative explanations, by integrating the FoA as a central component, the shared priority map framework also presents itself as a part of the three embedded components model (Cowan, 1999; Oberauer, 2002), which outlines the functional structure of the WM system. Thus, the shared priority map framework provides a comprehensive explanation of LAN embedded within the WM system and accounting for the presence of it for predominantly verbal and visual strategies.

### Functionality of LAN

Another consistent finding from our study was that we do not find any relation between LAN and memory performance – calling into question the functionality of LAN to memory. There are two possible explanations for these results. The first is that in this task participants show overall high memory performance; thus, we have many more trials with correct responses as compared to incorrect responses, leaving us with less data to inform on incorrect as compared to correct trials.

The second explanation comes from recent work by Bhanap and colleagues (2024) where they conducted a similar task but manipulated eye movements of participants during retrieval. That is, participants were required to look at either a congruent location with regards to the retrieved item or to an incongruent location. The premise was that if LAN aids in memory retrieval, congruent fixation would lead to a facilitation in memory performance. No such facilitation was found (but see Johansson & Johansson, 2014; Scholz et al., 2018). Bhanap and colleagues’ explanation was linked to the implemented set size of 3 word-pairs which is smaller compared to what had been used in previous studies (i.e. 16 in Scholz et al., 2018, and 24 by Johansson & Johansson, 2014). Specifically, they argued that the first probe during the WM test leads to the retrieval of the second word associated with it through a pairwise binding. The first probe also reactivates the associated location through visual attention. At a low set size, the competition in retrieving the word associated with the first probe is resolved in the focus of attention and the additional activation from LAN does not add anything more to the retrieval process. A similar situation may happen in our task, meaning we may not have been able to observe a significant facilitation in memory performance as also here, competition in retrieving the word associated with the first probe is resolved in the focus of attention.

To further test the idea of a functional relation between LAN and retrieval, we also looked at eye movements during the retention interval. As mentioned before, the idea was to test if the amount of LAN in the retention interval towards the location of a particular word pair was predictive of later memory performance for that word pair. We did not find such a relation. Instead, we found that participants show similar amounts of looking towards all three AOIs (plots are added in the supplementary materials). Similar findings were reported by Loaiza and Souza (2022), who showed that during retrieval participants fixated the empty locations at which information was encoded. However, there was no relation between the amount of LAN during the retention interval and the recall precision. This could signify that participants are engaging in the strategy instructed in that task for each of the three word-pairs and this is reflected in eye movements during the retention interval. A similar finding has been reported by Tremblay and colleagues (2006) for spatial rehearsal. The missing functionality of this behavior could again be explained by the low set size of three word-pairs, that enables participants to employ the strategy more or less similarly for all the word pairs, leading to no differentiation in the amount of eye movements to L1 and the performance in the memory task.

In our Experiment 1, we conducted an LTM test as well. Even though the major reason to run an LTM test was to ensure that participants engaged in visual imagery (Bartsch et al., 2018), and to replicate typical differential effects of rehearsal and imagery on WM and LTM, we also looked at the eye movement data. Previous studies on LAN have mainly studied it in in the context of LTM (Kinjo et al., 2020; Scholz et al., 2018), yet in these studies, the location was always unique to a low number of items. As in the present study every location was associated with 48 items, we expected to observe interference from different word pairs associated to the same context (Oberauer & Lin, 2017) and therefore, also less LAN compared to the WM test as well as compared to prior work. We did observe some amount of LAN indicating that even when the location information is not unique, some activation of location information survived. While understanding the relation between LAN and memory strength it is important to consider how memory strength is conceptualized across different memory systems. For WM, strength is often defined in terms of the precision or fidelity of the stored representation. From this perspective, LAN may reflect the change in the precision of traces, a process linked to fronto-parietal attentional control networks (D’Esposito & Postle, 2015). In contrast, the LTM literature typically defines memory strength in terms of the probability of successful recollection, a process largely supported by medial temporal lobe and hippocampal structures (Jeneson & Squire, 2012). Within this framework, LAN reflects the recollection of memory traces and not the precision of the trace. These differing interpretations underscore the importance of considering the underlying memory system when evaluating the role of LAN. In our study, during the LTM test phase, spatial locations were overloaded with multiple word pairs, meaning that no single location uniquely cued a specific memory trace. This lack of a one-to-one spatial association may explain why LAN modulations were not observed with varying LTM strength in the visual imagery conditions of PI and NoPI blocks.

### Implications for the relation between Articulatory Suppression and LAN

We observed the least amount of LAN for articulatory suppression. Previous work suggests that when people engage in AS it occupies their phonological loop and can lead to them using their visuo-spatial sketchpad instead to aid maintenance by activating visuo-spatial information. However, in our study we observed the least amount of LAN for articulatory suppression. In other words, we did not observe overwhelming evidence for the notion that the visuo-spatial sketchpad was activated when engaging in articulatory suppression. Instead, our findings are more in line with interference accounts of WM (Souza & Oberauer, 2018). We detail this in the following.

With regards to memory performance, we found no difference between WM and LTM performance under AS instructions in Experiment 1 and a decrease in memory performance with PI in Experiment 2. Specifically, in Experiment 1, the information verbally spoken during AS (“babibou”) could have interfered with the verbal information currently held in WM, thus we see worse WM performance as compared to other strategy conditions (Lewandowsky, 2008). What survives and is drawn upon in the WM test is the information that made it into LTM via the rapid formation of episodic representations at encoding (as suggested by Cowan, 2019). Thus, we do not see a difference between performance in the WM and LTM test. Additionally, the word pairs that are tested in WM are retested in LTM and the test itself may add stability to these survived representations (Roediger & Karpicke, 2006). In Experiment 2, in the PI session, relying on these LTM representations is less helpful, which leads to a further decrease in performance under AS – as neither LTM nor WM traces can be build-up reliably to drive performance.

## Conclusion

In this study, we show that location information is reactivated irrespective of the strategy people engaged in, whether it is visual imagery which is traditionally considered to be a visuo-spatial strategy or rehearsal which has been known to be a more verbal-auditory strategy. Additionally, by studying LAN under proper comparison conditions, we showcase that it is not exclusively driven by visual processing but rather reflects the retrieval of representations into the FoA, leading to shifts of attention toward the associated location, which manifests as LAN.

## Data Accessibility Statement

The preregistration, data and the analysis scripts can be accessed on the Open Science Framework (https://osf.io/c4rw3).

## Additional Files

The additional files for this article can be found as follows:

10.5334/joc.449.s1 Supplementary Materials. Table S1. Preregistration Deviations for Experiment 1.

10.5334/joc.449.s2 Supplementary Materials. Additional Plots and Analysis.

## Footnotes

Note that in parts of the literature the term “first probe” is used as synonym to the word “cue” and “second probe” as synonym for the term “probe”. Furthermore, the term “second probe” could be considered a “target” (here referred to as “positive” trial), “lure” (here also referred to as “lure” trial) or “foil” (here referred to as “new” trial).

## References

[1] Anderson, M. C., Bjork, E. L., & Bjork, R. A. (2000). Retrieval-induced forgetting: Evidence for a recall-specific mechanism. Psychonomic bulletin & review, 7, 522–530. 10.13758/BF03214366 11082860

[2] Atkins, A. S., Berman, M. G., Reuter-Lorenz, P. A., Lewis, R. L., & Jonides, J. (2011). Resolving semantic and proactive interference in memory over the short-term. Memory & cognition, 39, 806–817. 10.3758/s13421-011-0072-5 21327614 PMC4472387

[3] Atkinson, R. C., & Shiffrin, R. M. (1968). Human memory: A proposed system and its control processes. In Psychology of learning and motivation (Vol. 2, pp. 89–195). Academic press. 10.1016/S0079-7421(08)60422-3

[4] Awh, E., & Jonides, J. (2001). Overlapping mechanisms of attention and spatial working memory. Trends in cognitive sciences, 5(3), 119–126. 10.1016/S1364-6613(00)01593-X 11239812

[5] Baddeley, A. (1996). The fractionation of working memory. Proceedings of the National Academy of Sciences of the United States of America, 93(24), 13468–13472. 10.1073/pnas.93.24.13468 8942958 PMC33632

[6] Baddeley, A. (2000). The episodic buffer: a new component of working memory? Trends in cognitive sciences, 4(11), 417–423. 10.1016/S1364-6613(00)01538-2 11058819

[7] Bartsch, L. M., Loaiza, V. M., Jäncke, L., Oberauer, K., & Lewis-Peacock, J. A. (2019). Dissociating refreshing and elaboration and their impacts on memory. NeuroImage, 199, 585–597. 10.1016/j.neuroimage.2019.06.028 31207338 PMC11158115

[8] Bartsch, L. M., & Oberauer, K. (2023). The contribution of episodic LTM to WMfor bindings. Cognition, 231, 105330. 10.1016/j.cognition.2022.105330 36436446

[9] Bartsch, L. M., Singmann, H., & Oberauer, K. (2018). The effects of refreshing and elaboration on working memory performance, and their contributions to long-term memory formation. Memory and Cognition, 46(5), 796–808. 10.3758/s13421-018-0805-9 29557069

[10] Bartsch, L. M., Souza, A. S., & Oberauer, K. (2024). The benefits of memory control processes in working memory: Comparing effects of self-reported and instructed strategy use. Journal of Experimental Psychology: Learning, Memory, and Cognition. 10.1037/xlm0001370 38934928

[11] Bhanap, R., Oberauer, K., & Rosner, A. (2025). Investigating retrieval strategies in an associative recognition test in working memory: Evidence from eye movements. Cognition, 263, 106199. 10.1016/j.cognition.2025.106199 40483814

[12] Bhanap, R., Oberauer, K., & Rosner, A. (2024, September 12). The role of eye movements and covert shifts of attention in working memory retrieval. 10.31234/osf.io/5d73r PMC13269553 42298274

[13] Bürkner, P. C. (2017). brms: An R package for Bayesian multilevel models using Stan. Journal of Statistical Software, 80(1). 10.18637/jss.v080.i01

[14] Camos, V., & Barrouillet, P. (2014). Attentional and non-attentional systems in the maintenance of verbal information in working memory: the executive and phonological loops. Frontiers in human neuroscience, 8, 900. 10.3389/fnhum.2014.00900 25426049 PMC4224087

[15] Chiquet, S., Martarelli, C. S., & Mast, F. W. (2021). Eye movements to absent objects during mental imagery and visual memory in immersive virtual reality. Virtual Reality, 25(3), 655–667. 10.1007/s10055-020-00478-y

[16] Cowan, N. (1999). An embedded-processes model of working memory. Models of working memory: Mechanisms of active maintenance and executive control, 20(506), 1013–1019. 10.1017/CBO9781139174909.006

[17] Cowan, N. (2017). The many faces of working memory and short-term storage. Psychonomic bulletin & review, 24, 1158–1170. 10.3758/s13423-016-1191-6 27896630

[18] Cowan, N. (2019). Short-term memory based on activated long-term memory: A review in response to Norris (2017). 10.1037/bul0000199 PMC6650160 31328941

[19] Craik, F. I. M., & Lockhart, R. S. (1972). Levels of processing: A framework for memory research. Journal of Verbal Learning and Verbal Behavior, 11, 671–684. 10.1016/S0022-5371(72)80001-X

[20] Craik, F. I., & Tulving, E. (1975). Depth of processing and the retention of words in episodic memory. Journal of experimental Psychology: general, 104(3), 268. 10.1037/0096-3445.104.3.268

[21] Crannell, C. W., & Parrish, J. M. (1957). A comparison of immediate memory span for digits, letters, and words. The Journal of Psychology, 44(2), 319–327. 10.1080/00223980.1957.9713089

[22] D’Esposito, M., & Postle, B. R. (2015). The cognitive neuroscience of working memory. Annual review of psychology, 66(1), 115–142. 10.1146/annurev-psych-010814-015031 PMC4374359 25251486

[23] Dunlosky, J., & Hertzog, C. (2001). Measuring strategy production during associative learning: The relative utility of concurrent versus retrospective reports. Memory & Cognition, 29(2), 247–253. 10.3758/BF03194918 11352207

[24] Ferreira, F., Apel, J., & Henderson, J. M. (2008). Taking a new look at looking at nothing. Trends in cognitive sciences, 12(11), 405–410. 10.1016/j.tics.2008.07.007 18805041

[25] Greene, R. L. (1987). Effects of maintenance rehearsal on human memory. Psychological Bulletin, 102(3), 403. 10.1037/0033-2909.102.3.403

[26] Guérard, K., Morey, C. C., Lagacé, S., & Tremblay, S. (2013). Asymmetric binding in serial memory for verbal and spatial information. Memory & cognition, 41, 378–391. 10.3758/s13421-012-0275-4 23254536

[27] Guérard, K., Tremblay, S., & Saint-Aubin, J. (2009). Short article: Similarity and binding in memory: Bound to be detrimental. Quarterly Journal of Experimental Psychology, 62(1), 26–32. 10.1080/17470210802215277 18622886

[28] Gurtner, L. M., Hartmann, M., & Mast, F. W. (2021). Eye movements during visual imagery and perception show spatial correspondence but have unique temporal signatures. Cognition, 210. 10.1016/j.cognition.2021.104597 33508576

[29] Hedge, C., & Leonards, U. (2013). Using eye movements to explore switch costs in working memory. Journal of Vision, 13(4), 18–18. 10.1167/13.4.18 23525134

[30] Hedge, C., Oberauer, K., & Leonards, U. (2015). Selection in spatial working memory is independent of perceptual selective attention, but they interact in a shared spatial priority map. Attention, Perception, & Psychophysics, 77, 2653–2668. 10.3758/s13414-015-0976-4 PMC4644201 26341873

[31] Hoover, M. A., & Richardson, D. C. (2008). When facts go down the rabbit hole: Contrasting features and objecthood as indexes to memory. Cognition, 108(2), 533–542. 10.1016/j.cognition.2008.02.011 18423431

[32] Jahn, G., & Braatz, J. (2014). Memory indexing of sequential symptom processing in diagnostic reasoning. Cognitive psychology, 68, 59–97. 10.1016/j.cogpsych.2013.11.002 24316414

[33] Jeneson, A., & Squire, L. R. (2012). Working memory, long-term memory, and medial temporal lobe function. Learning & memory, 19(1), 15–25. 10.1101/lm.024018.111 22180053 PMC3246590

[34] Johansson, R., Holsanova, J., Dewhurst, R., & Holmqvist, K. (2012). Eye movements during scene recollection have a functional role, but they are not reinstatements of those produced during encoding. Journal of Experimental Psychology: Human Perception and Performance, 38(5), 1289–1314. 10.1037/a0026585 22201467

[35] Johansson, R., & Johansson, M. (2014). Look Here, Eye Movements Play a Functional Role in Memory Retrieval. Psychological Science, 25(1), 236–242. 10.1177/0956797613498260 24166856

[36] Johansson, R., Nyström, M., Dewhurst, R., & Johansson, M. (2022). Eye-movement replay supports episodic remembering. Proceedings of the Royal Society B, 289(1977), 20220964. 10.1098/rspb.2022.0964 35703049 PMC9198773

[37] Kahana, M. J. (2002). Associative symmetry and memory theory. Memory & cognition, 30(6), 823–840. 10.3758/BF03195769 12450087

[38] Kinjo, H., Fooken, J., & Spering, M. (2020). Do eye movements enhance visual memory retrieval? Vision Research, 176, 80–90. 10.1016/j.visres.2020.07.013 32827879

[39] Klichowicz, A., Lippoldt, D. E., Rosner, A., & Krems, J. F. (2021). Information stored in memory affects abductive reasoning. Psychological Research, 85(8), 3119–3133. 10.1007/s00426-020-01460-8 33428007 PMC8476388

[40] Klichowicz, A., Strehlau, S., Baumann, M. R., Krems, J. F., & Rosner, A. (2020). Tracing current explanations in memory: A process analysis based on eye-tracking. Quarterly Journal of Experimental Psychology, 73(10), 1703–1717. 10.1177/1747021820922509 32338577

[41] Kosslyn, S. M., Thompson, W. L., & Ganis, G. (2006). The case for mental imagery. Oxford University Press. 10.1093/acprof:oso/9780195179088.001.0001

[42] Kubinec, R. (2023). Ordered Beta Regression: A Parsimonious, Well-Fitting Model for Continuous Data with Lower and Upper Bounds. Political Analysis, 31(4), 519–536. 10.1017/pan.2022.20

[43] Kumcu, A., & Thompson, R. L. (2020). Less imageable words lead to more looks to blank locations during memory retrieval. Psychological Research, 84(3), 667–684. 10.1007/s00426-018-1084-6 30173279 PMC7109172

[44] Laeng, B., Bloem, I. M., D’Ascenzo, S., & Tommasi, L. (2014). Scrutinizing visual images: The role of gaze in mental imagery and memory. Cognition, 131(2), 263–283. 10.1016/j.cognition.2014.01.003 24561190

[45] Laeng, B., & Teodorescu, D. S. (2002). Eye scanpaths during visual imagery reenact those of perception of the same visual scene. Cognitive science, 26(2), 207–231. 10.1207/s15516709cog2602_3

[46] Lewandowsky, S. (2008). Short-Term Memory: New Data and a Model Stephan Lewandowsky University of Western Australia and Simon Farrell University of Bristol. Learning, 49. 10.1016/S0079-7421(08)00001-7

[47] Loaiza, V. M., & Souza, A. S. (2022). The eyes don’t have it: Eye movements are unlikely to reflect refreshing in working memory. PloS one, 17(7), e0271116. 10.1371/journal.pone.0271116 35834590 PMC9282440

[48] Luck, S. J., & Vogel, E. K. (1997). The capacity of visual working memory for features and conjunctions. Nature, 390(6657), 279–281. 10.1038/36846 9384378

[49] Marsh, J. E., Hurlstone, M. J., Marois, A., Ball, L. J., Moore, S. B., Vachon, F., Schlittmeier, S. J., Röer, J. P., Buchner, A., Aust, F., & Bell, R. (2024). Changing-state irrelevant speech disrupts visual–verbal but not visual–spatial serial recall. Journal of Experimental Psychology: Learning, Memory, and Cognition. Advance online publication. 10.1037/xlm0001360 38913725

[50] Martarelli, C. S., & Mast, F. W. (2013). Eye movements during long-term pictorial recall. Psychological Research, 77(3), 303–309. 10.1007/s00426-012-0439-7 22610303

[51] Mazuryk, G. F., & Lockhart, R. S. (1974). Negative recency and levels of processing in free recall. Canadian Journal of Psychology/Revue Canadienne de Psychologie, 28(1), 114–123. 10.1037/h0081971

[52] Meiser, T., & Klauer, K. C. (1999). Working memory and changing-state hypothesis. Journal of Experimental Psychology: Learning, Memory, and Cognition, 25, 1272–1299. 10.1037/0278-7393.25.5.1272

[53] Oberauer, K. (2002). Access to information in working memory: exploring the focus of attention. Journal of Experimental Psychology: Learning, Memory, and Cognition, 28(3), 411. 10.1037//0278-7393.28.3.411 12018494

[54] Oberauer, K., & Lin, H. Y. (2017). An interference model of visual working memory. Psychological Review, 124(1), 21–59. 10.1037/rev0000044 27869455

[55] Pearson, D. G., Logie, R. H., & Gilhooly, K. J. (1999). Verbal representations and spatial manipulation during mental synthesis. European Journal of Cognitive Psychology, 11(3), 295–314. 10.1080/713752317

[56] Peterson, M. J., Thomas, J. E., & Johnson, H. (1977). Imagery, rehearsal, and the compatibility of input-output tasks. Memory & Cognition, 5(4), 415–422. 10.3758/BF03197380 24203008

[57] Renkewitz, F., & Jahn, G. (2012). Memory indexing: a novel method for tracing memory processes in complex cognitive tasks. Journal of Experimental Psychology: Learning, Memory, and Cognition, 38(6), 1622. 10.1037/a0028073 22545615

[58] Richardson, D. C., & Spivey, M. J. (2000). Representation, space and Hollywood Squares: Looking at things that aren’t there anymore. Cognition, 76(3), 269–295. 10.1016/S0010-0277(00)00084-6 10913578

[59] Roediger, H. L., & Karpicke, J. D. (2006). The Power of Testing Memory: Basic Research and Implications for Educational Practice. Perspectives on Psychological Science, 1(3), 181–210. 10.1111/j.1745-6916.2006.00012.x 26151629

[60] Rosner, A., Pantoja, M., & Bhanap, R. (in prep.). Eye Movements Reflect the Strength of Activation in Working Memory.

[61] Rosner, A., Schaffner, M., & von Helversen, B. (2022). When the eyes have it and when not: How multiple sources of activation combine to guide eye movements during multiattribute decision making. Journal of Experimental Psychology: General, 151(6), 1394. 10.1037/xge0000833 34748360

[62] Rosner, A., & von Helversen, B. (2019). Memory shapes judgments: Tracing how memory biases judgments by inducing the retrieval of exemplars. Cognition, 190, 165–169. 10.1016/j.cognition.2019.05.004 31100546

[63] Scholz, A., Klichowicz, A., & Krems, J. F. (2018). Covert shifts of attention can account for the functional role of “eye movements to nothing.” Memory and Cognition, 46(2), 230–243. 10.3758/s13421-017-0760-x 28975576

[64] Scholz, A., Krems, J. F., & Jahn, G. (2017). Watching diagnoses develop: Eye movements reveal symptom processing during diagnostic reasoning. Psychonomic Bulletin & Review, 24, 1398–1412. 10.3758/s13423-017-1294-8 28444634

[65] Scholz, A., Mehlhorn, K., Bocklisch, F., & Krems, J. (2011). Looking at nothing diminishes with practice. In Proceedings of the annual meeting of the cognitive science society (Vol. 33, No. 33).

[66] Scholz, A., Mehlhorn, K., & Krems, J. F. (2016). Listen up, eye movements play a role in verbal memory retrieval. Psychological research, 80, 149–158. 10.1007/s00426-014-0639-4 25527078

[67] Shah, P., & Miyake, A. (1996). The separability of working memory resources for spatial thinking and language processing: an individual differences approach. Journal of experimental psychology: General, 125(1), 4. 10.1037/0096-3445.125.1.4 8851737

[68] Shaughnessy, J. J. (1981). Memory monitoring accuracy and modification of rehearsal strategies. Journal of Verbal Learning and Verbal Behavior, 20(2), 216–230. 10.1016/S0022-5371(81)90389-3

[69] Souza, A. S., & Oberauer, K. (2018). Does articulatory rehearsal help immediate serial recall? Cognitive Psychology, 107, 1–21. 10.1016/j.cogpsych.2018.09.002 30292953

[70] Souza, A. S., & Oberauer, K. (2020). No Evidence That Articulatory Rehearsal Improves Complex Span Performance. Journal of Cognition, 3(1), 1–25. 10.5334/joc.103 32435749 PMC7227395

[71] Tan, L., & Ward, G. (2008). Rehearsal in immediate serial recall. Psychonomic bulletin & review, 15(3), 535–542. 10.3758/PBR.15.3.535 18567251

[72] Thomas, N. J. (2009). Visual imagery and consciousness. Encyclopedia of consciousness, 2, 445–457. 10.1016/B978-012373873-8.00083-9

[73] Theeuwes, J., Belopolsky, A., & Olivers, C. N. (2009). Interactions between working memory, attention and eye movements. Acta psychologica, 132(2), 106–114. 10.1016/j.actpsy.2009.01.005 19233340

[74] Tremblay, S., Saint-Aubin, J., & Jalbert, A. (2006). Rehearsal in serial memory for visual-spatial information: Evidence from eye movements. Psychonomic bulletin & review, 13(3), 452–457. 10.3758/BF03193869 17048730

[75] Tulving, E. (1983). Elements of episodic memory. Canadian Psychology, 26(3), 351.

[76] Umar, H., Mast, F. W., Cacchione, T., & Martarelli, C. S. (2021). The prioritization of visuo-spatial associations during mental imagery. Cognitive Processing, 22(2), 227–237. 10.1007/s10339-020-01010-5 33404898

[77] Vieira, J., Rosner, A., Bhanap, R., & Souza, A. S. (2023, March). Looking at nothing and memory strength in working memory. Paper presented at the 17th National Meeting of the Portuguese Association of Experimental Psychology, Lisbon. 10.13140/RG.2.2.29062.65600

[78] Wilhelm, O., Hildebrandt, A., & Oberauer, K. (2013). What is working memory capacity, and how can we measure it? Frontiers in Psychology, 4, 433. 10.3389/fpsyg.2013.00433 23898309 PMC3721021

[79] Willroth, E. C., & Atherton, O. E. (2024). Best Laid Plans: A Guide to Reporting Preregistration Deviations. Advances in Methods and Practices in Psychological Science, 7(1). 10.1177/25152459231213802

[80] Wynn, J. S., Liu, Z. X., & Ryan, J. D. (2022). Neural correlates of subsequent memory-related gaze reinstatement. Journal of cognitive neuroscience, 34(9), 1547–1562. 10.1162/jocn_a_01761 34272959

