# Supplementary material for: Tracking Reactivation of Location Information during Memory Strategies: Insights from Eye Movements
Source: J Cogn. 2025 Jul 2;8(1):38. doi: 10.5334/joc.449 (PMC12227092; doi:10.5334/joc.449)
Supplement: Supplementary Materials. — Table S1. Preregistration Deviations for Experiment 1. [file joc-8-1-449-s1.pdf]

Table S1

Preregistration Deviations for Experiment 1

**Deviations**					
#	Details		Original Wording	Deviation Description	Reader Impact
1	Type	Study Design	*During the encoding phase, participants will be presented sequentially with four-word pairs in four different locations.*<br><br>*Here, the screen will turn into a particular colour for 2 seconds, each colour indicates the strategy that the participants must engage (green: visual imagery, blue: articulatory rehearsal, red: articulatory suppression)*	*We conducted a pilot study with the same study design as mentioned in the preregistration. Out of the 24 participants that we collected, we had to exclude 11 for working memory performance that was less than chance. One possible explanation for such a low level of performance could be that the task was too difficult for the participants. Thus, for the current study we reduced the set size from four to three word-pairs to make the task easier for participants. To reduce the cognitive load even further, we explicitly mention on the screen which strategy they have to engage in along with the colored screen.*	*We derived the study design from a previous study from Bhanap and colleagues (2024) where a set size of four word-pairs was used for the task. We adapted the same task for our study and added color coded strategy instructions. For participants, in addition to storing and retrieving the word pairs, they also had to remember and retrieve the strategy associated with each color. Overall, this could have increased the difficulty level of the task, thus leading to such poor performance.*
	Reason	Other (Please Explain)			
	Timing	Other (Please Explain)			
2	Type	Study Design	*The instructions which strategy to engage in are only presented to participants after encoding of the list, at the beginning of the retention phase*	*We conducted a pilot study where in the LTM memory phase we did not observe a benefit of visual imagery on the LTM memory performance. It was essential to observe this benefit to ensure that*	*Our initial design plan to instruct only after participants encode all the word pairs was motivated by the idea that we can keep a similar encoding phase for all the strategy conditions. However, this decision proved*
	Reason	New knowledge			
	Timing	Other (Please Explain)			

				participants did engage in visual imagery during the working memory phase based on the results from previous work. One possible explanation could be that as the instruction to engage in visual imagery occurred only after the word pairs, it may have been difficult for the participants to retrieve all the word pairs and form mental images within the retention interval. Thus, we shifted the instructions before encoding so that participants can start forming mental images as soon as they see the word pair, helping with the creation of them.	*deterrent towards the actual use of the strategy. Therefore, the readers should consider for their future research which design idea is more central to the experiment and make design plans accordingly.*
