# Supplementary material for: Tracking Reactivation of Location Information during Memory Strategies: Insights from Eye Movements
Source: J Cogn. 2025 Jul 2;8(1):38. doi: 10.5334/joc.449 (PMC12227092; doi:10.5334/joc.449)
Supplement: Supplementary Materials. — Additional Plots and Analysis. [file joc-8-1-449-s2.pdf]

Supplementary Materials: Tracking Reactivation of Location Information during Memory Maintenance Strategies: Insights from Eye Movements.

1. **LAN for Inter Word Interval and Second Probe**

As reported by Bhanap and colleagues (2024), the amount of LAN that is observed throughout a retrieval phase can change across the timeline. Accordingly, we formalized this in an analysis to see if Visual Imagery lead to more LAN during inter word interval and second probe when the strength of LAN is the highest (Figure S1 and S2). We ran the ordered beta regression models with ordbetareg (Kubinec, 2023) package in R with RStudio, but we restricted the data to the probe interval till the end of the second probe. We did not see any credible difference in the amount of LAN observed across the strategies even for this duration (Table S1 and S2). The same is observed for both the experiments. Thus, the amount

Figure S1

Fixation Proportion for L1 across strategies and the two phases for Inter Word Interval and Second Probe. The error bars indicate 95% within subject confidence intervals.

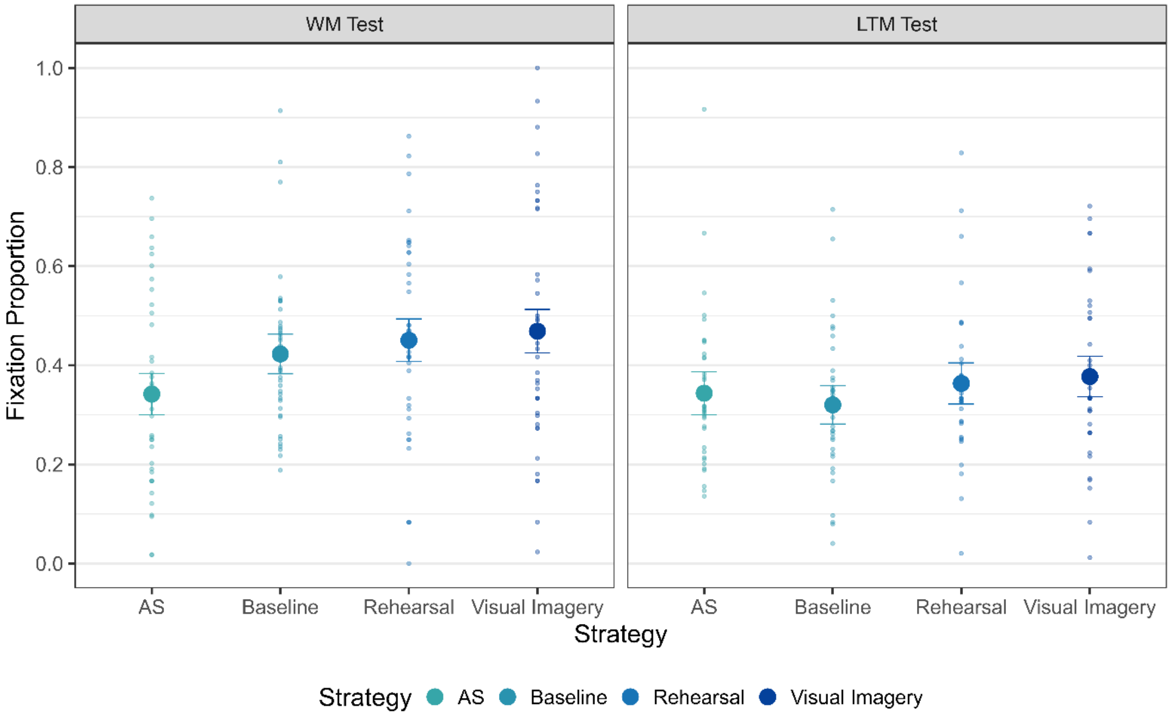

of LAN observed does not differ across strategies.

Table S1

Bayes Factor (BF_{10}) for the pairwise comparisons between strategies for fixation proportion to L1 for both WM and LTM Test for the duration of interval and second probe.

Strategy	Baseline		Rehearsal		Visual Imagery	
	WM test	LTM test	WM test	LTM test	WM test	LTM test
AS	1.14	0.06	0.25	0.05	0.60	0.05
Baseline			0.05	0.16	0.11	0.08
Rehearsal					0.04	0.07

Figure S2

Fixation Proportion towards L1 during Inter Word Interval and Second Probe across two phases. The error bars indicate 95% within subject confidence intervals.

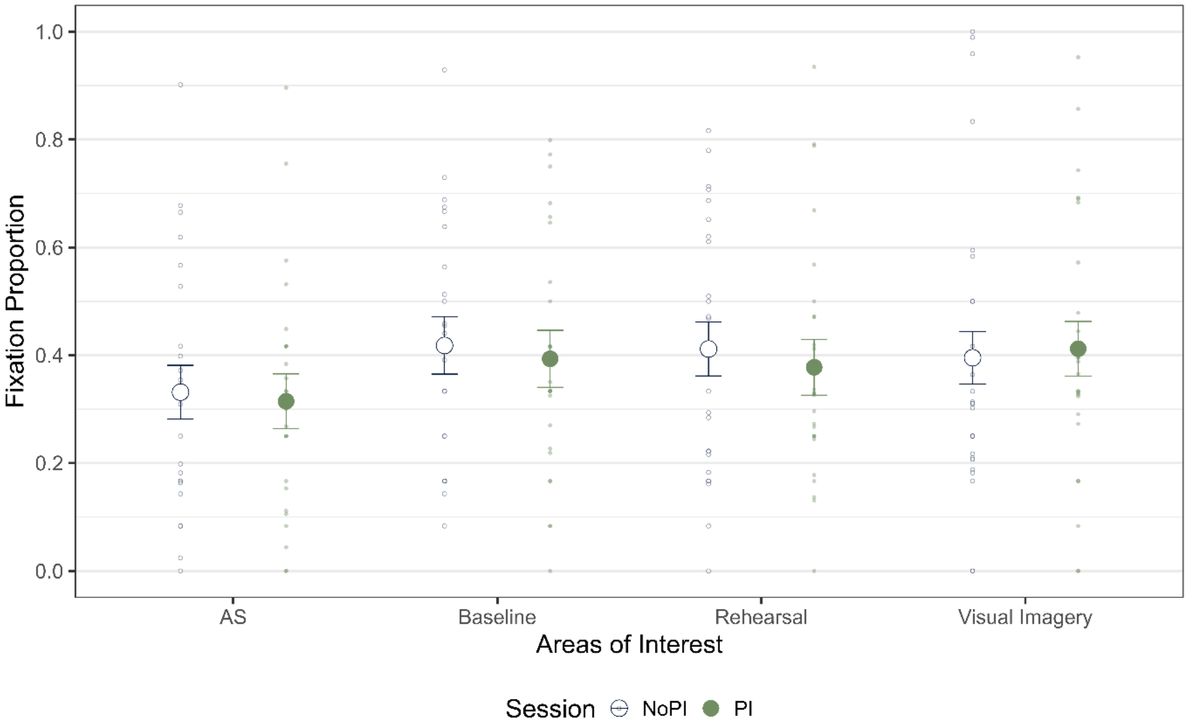

Table S2

Bayes Factor (BF10) for a main effect of strategies for fixation proportion to L1 for both PI and No PI session for the duration of interval and second probe.

Strategy	Baseline		Rehearsal		Visual Imagery	
	No PI	PI	No PI	PI	No PI	PI
AS	0.22	0.11	0.12	0.17	0.11	0.12

Baseline	0.08	0.07	0.10	0.08
Rehearsal			0.08	0.09

2. Eye Movement Analysis during Retention Interval

We ran the analysis to investigate the functionality of LAN across strategies – that is, whether the amount of LAN observed in retention interval was affected by the correctness of the trial differentially across strategies (Figure S3 and S4). The analysis has been reported in the main manuscript (Table 5 and Table 11), the plots for the same are added below. The results show that there is evidence against a main effect of correctness for all strategies. The same is observed for both Experiment 1 and 2.

Figure S2

Fixation Proportion to L1 during retention interval across strategies and correctness on the memory task for Experiment 1. The error bars indicate 95% within subject confidence intervals.

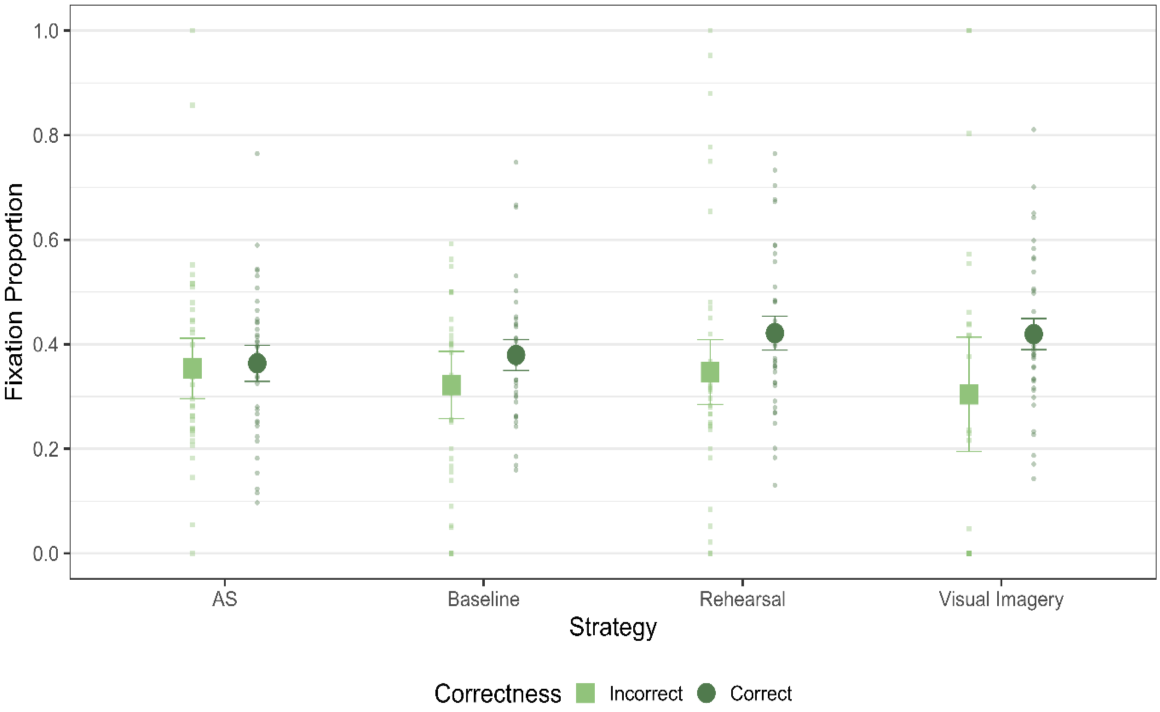

Figure S4

Fixation Proportion towards L1 during Retention Interval across the two phases and correctness of the trial for Experiment 2. The error bars indicate 95% within subject confidence intervals.

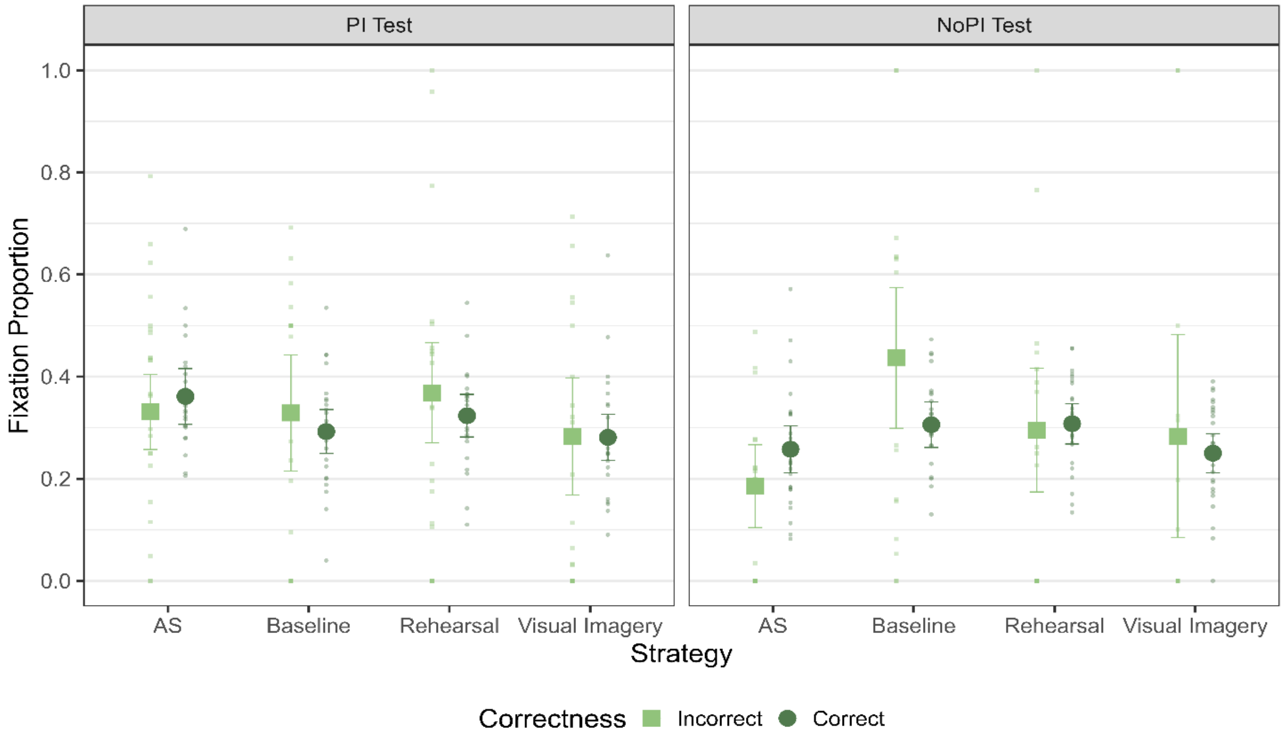

Additionally, we looked at whether participants looked at all three AOIs equally while engaging in different strategies. Especially, whether they looked more at the AOIs during visual imagery, thus, hinting towards the reactivation of location when participants engaged in the strategy. We plotted the fixation proportion to three AOIs during the retention interval (Figure S5 and S6). However, visual inspection of the plots showed that they looked equally at all three AOIs for all the strategies. The same was observed for both the experiments. Thus, there was no difference in the reactivation of location information when participants engaged in the strategy instructed.

Figure S5

Fixation Proportion to the three AOIs during retention interval for Experiment 1. The error bars indicate 95% within subject confidence intervals.

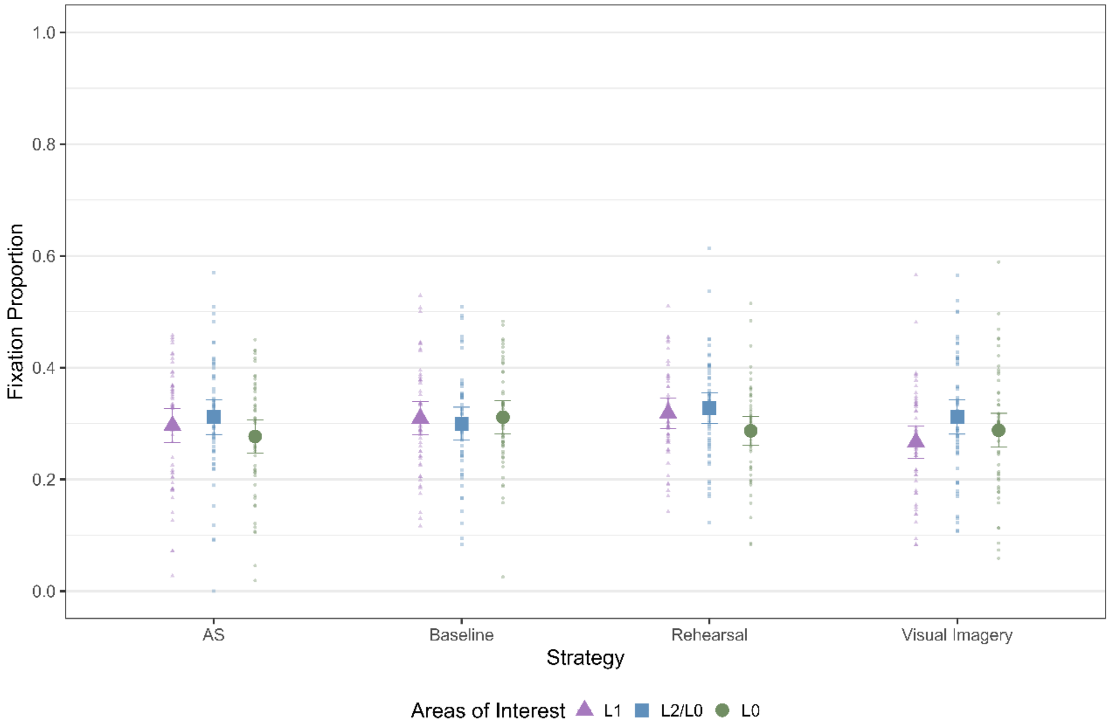

Figure S6

Fixation Proportion towards each AOI during Retention Interval for Experiment 2 for both PI and NoPI condition. The error bars indicate 95% within subject confidence intervals.

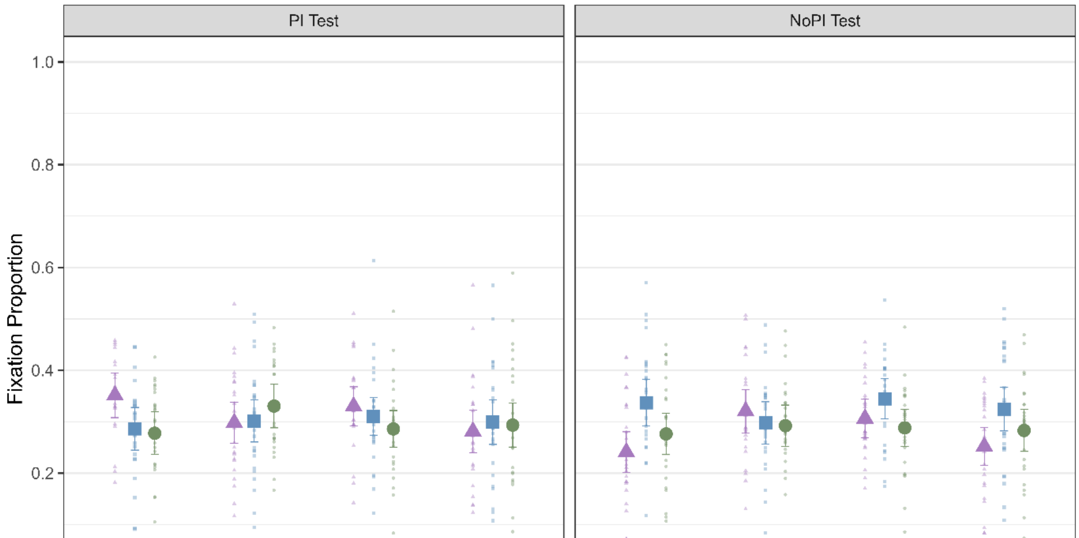

3. Block Wise Analysis: Experiment 2

3.1 WM Performance

The effect of proactive interference is known to accumulate over trials (Bunting, 2006; Oberauer & Bartsch, 2023). To examine this, we analyzed the WM performance across the experiment by dividing the trials into four blocks, each consisting of 12 trials. Like mentioned in the main manuscript, we estimated dprime across the two PI sessions, blocks and strategies from the Bayesian GLM based on the accuracy results. We compared each strategy condition and the effect of PI through pairwise comparison using the *emmeans* package.

First, we analyzed if the WM performance decreased over trials for the PI condition as compared to for the NoPI condition for different strategy conditions. As shown in Figure S7 and Table S3, there is a slight decrease in WM performance for AS from Block 1 to 4, however, there is inconclusive evidence for a main effect of block. In Visual Imagery, there is some decrease in WM performance from Block 2 to 3, however, this decrease is inconclusive. Thus, there is some decrease in WM performance, but the results remain largely ambiguous.

Figure S7

dprime across the four blocks for all strategies. The error bars indicate 95% highest posterior density intervals.

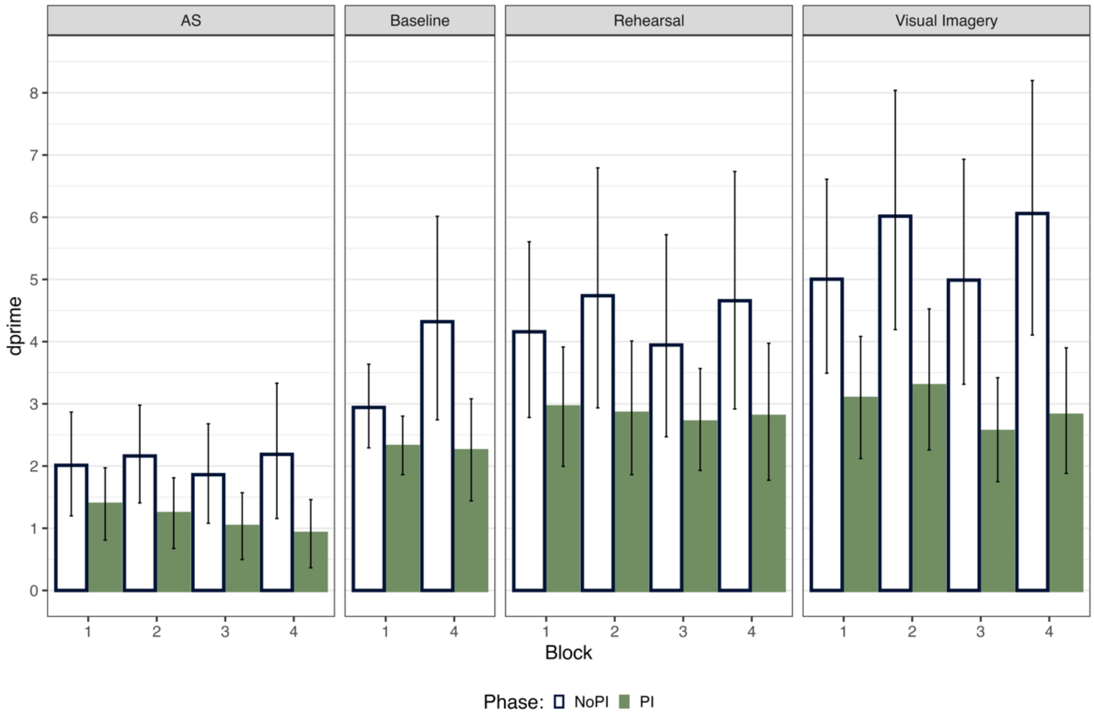

Table S3

Bayes Factor (BF10) for main effect of block across all strategies and the two PI conditions.

Strategy	AS		Baseline		Rehearsal		Visual Imagery	
	No PI	PI	No PI	PI	No PI	PI	No PI	PI
1 - 2	0.25	0.28	-	-	0.41	0.32	0.59	0.34
1 - 3	0.25	0.41	-	-	0.31	0.30	0.33	0.44
1 – 4	0.29	0.60	1.40	0.21	0.38	0.32	0.60	0.33
2 – 3	0.21	0.20	-	-	0.35	0.23	0.43	0.44
2 – 4	0.21	0.27	-	-	0.31	0.25	0.31	0.31
3 – 4	0.24	0.19	-	-	0.33	0.22	0.43	0.23

3.2 Eye Movement Analysis

We then investigated whether the decline in WM performance over the course of the trials influenced LAN to L1. To explore this, we assessed fixation proportion to L1 across all probe types for both the PI and NoPI conditions. As shown in Figure S8 and Table S4, our analysis revealed no evidence of a main effect of block when comparing each block to the others. Therefore, we found no credible change in LAN over the course of the trials due to the PI. Next, we examined whether the strategy condition affected the impact of PI on LAN over the trials (Figure S9 and Table S5). We observed a similar trend: there was no substantial evidence for a main effect of block in most cases, except for a few instances where the evidence was inconclusive. Finally, we assessed whether the buildup of PI interacted with trial correctness to influence LAN (Figures S10 and S11, and Table S6). With the exception of Block 1, where we observed higher LAN for correct trials compared to incorrect trials for AS, the evidence in other cases was either against or inconclusive regarding the main effect of correctness across strategies and blocks. Thus, proactive interference did not have an effect on the amount of LAN over the course of trials.

Figure S8

Fixation Proportion to L1 for all probe types, for both PI and NoPI block, across 4 blocks. Each block consists of 12 trials. The error bars indicate 95% within subject confidence intervals

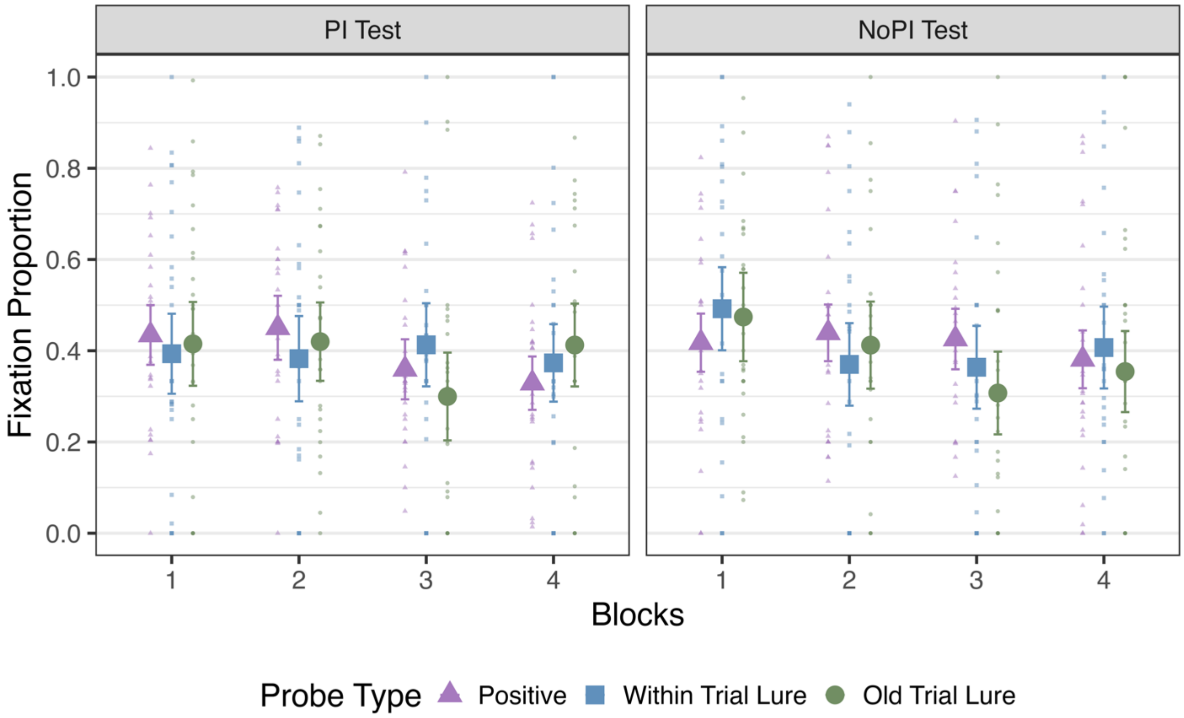

Table S4

Bayes Factor (BF10) for comparison of fixation proportion to L1 across blocks, for all probe types and the PI conditions.

Probe Type	Positive		Within Trial Lure		Old Trial Lure	
	No PI	PI	No PI	PI	No PI	PI
1 - 2	0.03	0.04	0.12	0.05	0.06	0.05
1 - 3	0.04	0.07	0.16	0.05	1.08	0.37
1 – 4	0.04	0.20	0.07	0.08	0.15	0.05
2 – 3	0.04	0.14	0.06	0.07	0.25	0.19
2 - 4	0.04	0.51	0.05	0.05	0.07	0.04
3 - 4	0.03	0.04	0.06	0.12	0.08	0.18

Figure S9

Fixation Proportion to L1 across strategies and NoPI and PI condition. The error bars

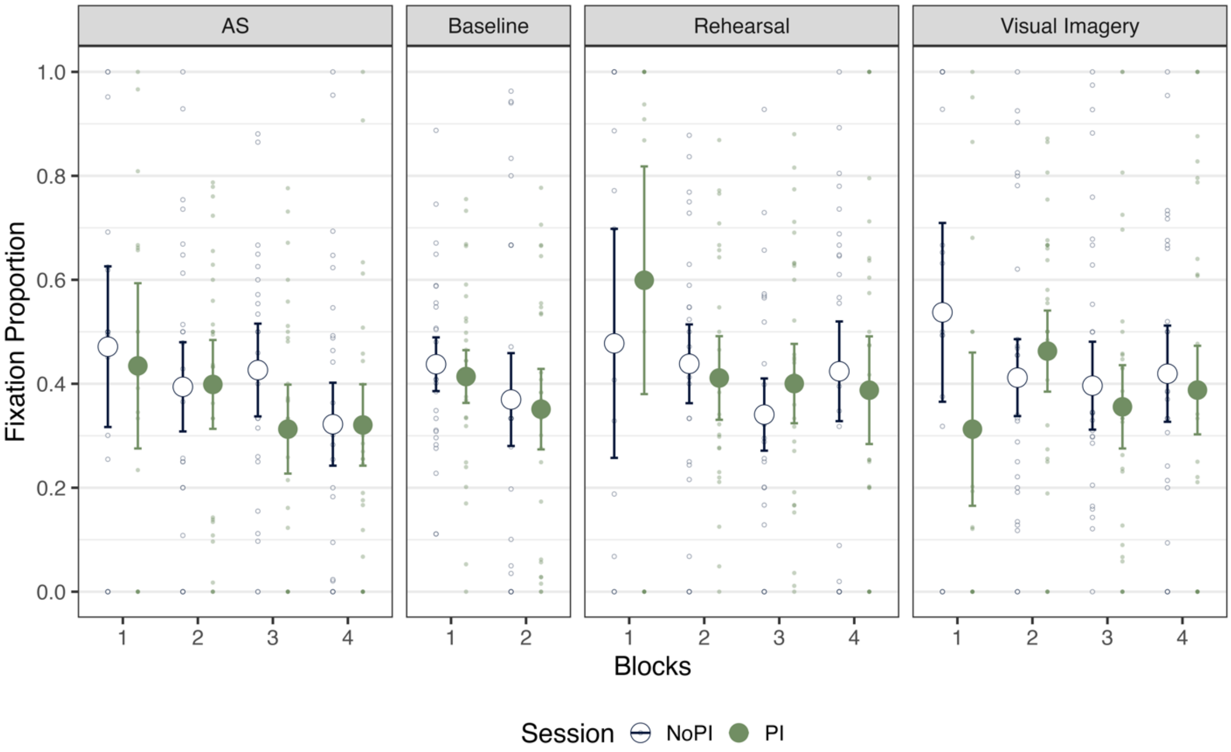

indicate 95% within subject confidence intervals.

Table S5

Bayes Factor (BF10) for comparison of fixation proportion to L1 across blocks, for all strategies and the PI conditions.

Strategy	AS		Baseline		Rehearsal		Visual Imagery	
	No PI	PI	No PI	PI	No PI	PI	No PI	PI
1 - 2	0.08	0.07	-	-	0.31	0.72	0.10	0.10
1 - 3	0.15	0.11	-	-	0.68	0.69	0.08	0.07
1 – 4	0.18	0.11	0.04	0.10	0.57	2.21	0.08	0.09
2 – 3	0.08	0.08	-	-	0.05	0.05	0.08	0.10
2 – 4	0.11	0.11	-	-	0.05	0.07	0.07	0.05
3 – 4	0.05	0.05	-	-	0.04	0.08	0.05	0.07

Figure S10

Fixation Proportion to L1 for correct and incorrect trials, across all strategies over the experiment for the PI condition. The four blocks consist of 12 trials each. The first block consists of 9 trials from Baseline Block and only 3 trials from one of the strategy condition. The error bars indicate 95% within subject confidence intervals.

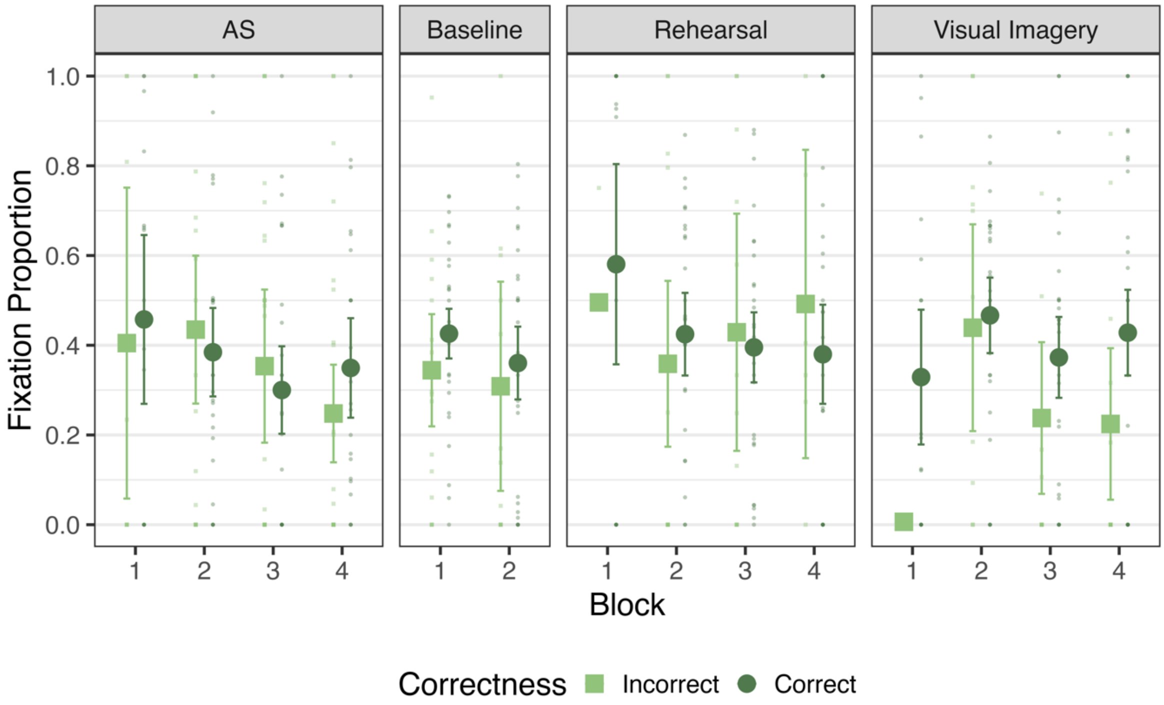

Table S6

Bayes Factor for Main Effect of Correctness across blocks, strategies for both the PI conditions.

Strategy	AS		Baseline		Rehearsal		Visual Imagery	
	No PI	PI	No PI	PI	No PI	PI	No PI	PI
1	4.06	0.14	0.08	0.16	0.62	0.43	0.37	0.53
2	0.08	0.07	-	-	0.20	0.10	0.11	0.08
3	0.07	0.13	-	-	0.13	0.09	0.52	0.85
4	0.08	0.08	0.08	0.10	1.14	0.12	0.10	0.13

Figure S11

Fixation Proportion to L1 for correct and incorrect trials, across all strategies over the experiment. The four blocks consist of 12 trials each. The first block consists of 9 trials from Baseline Block and only 3 trials from one of the strategy condition. The error bars indicate 95% within subject confidence intervals.

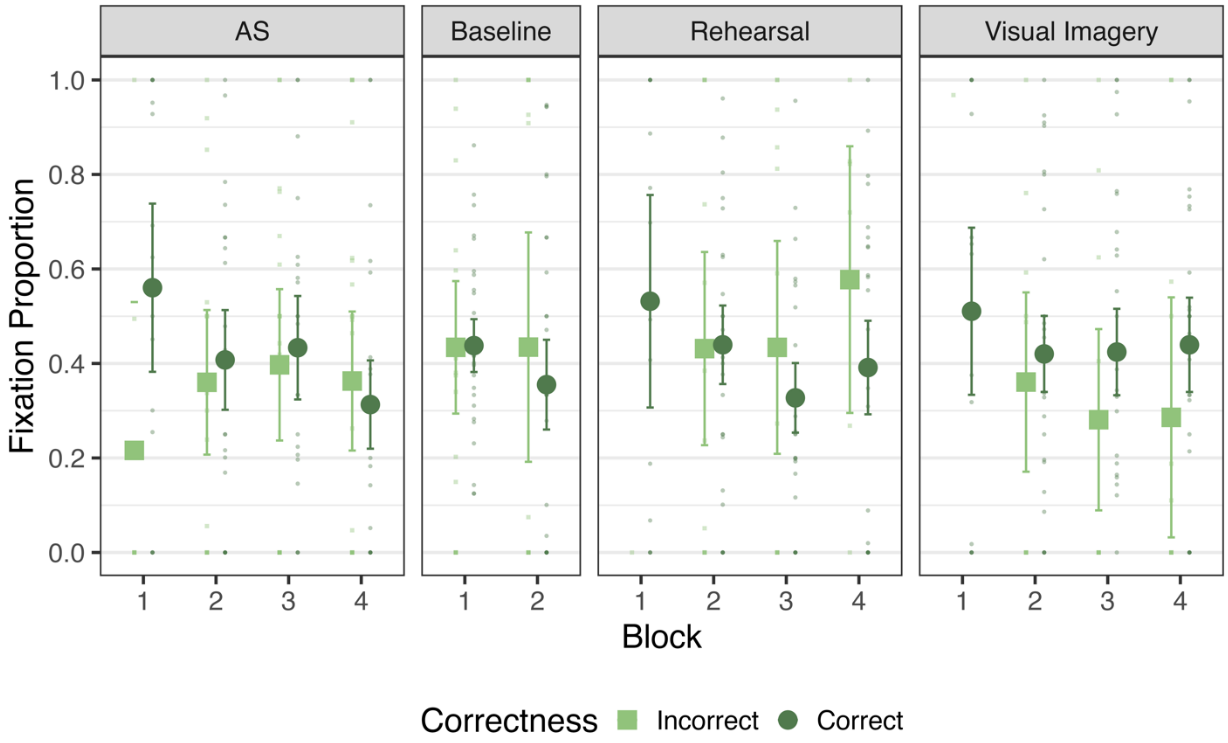

4. **LAN analysis across Frames**

Additionally, we looked at LAN to L1 across the time course of the retrieval phase by looking at the fixation proportion during each event: first probe, inter word interval, second probe, beep and response. Henceforth, these events are referred to as frames. The figures S12, S13, S14, S15 display fixation proportion to L1 across all strategies, AOIs and frames. In the WM test, we observe higher LAN to L1 for Visual Imagery as compared to AS during inter word interval and second probe, however, Baseline, Rehearsal and Visual Imagery do not differ from each other (Figure S12). For AS, Rehearsal, and Visual Imagery, fixation proportion is lower during the Beep and Response Screen phases, likely reflecting preparation for the strategy-use question, which appears at the center of the screen (Nikolov et al., 2024). A similar trend is observed in the LTM test, there is evidence against a

difference between the amount of LAN observed for different strategies at all the different timepoints (Figure S13).

Figure S12

Fixation Proportion to the three AOIs across frames and strategies for WM test. The error bars indicate 95% within subject confidence intervals. AS: Articulatory Suppression, B: Baseline, R: Rehearsal, VI: Visual Imagery.

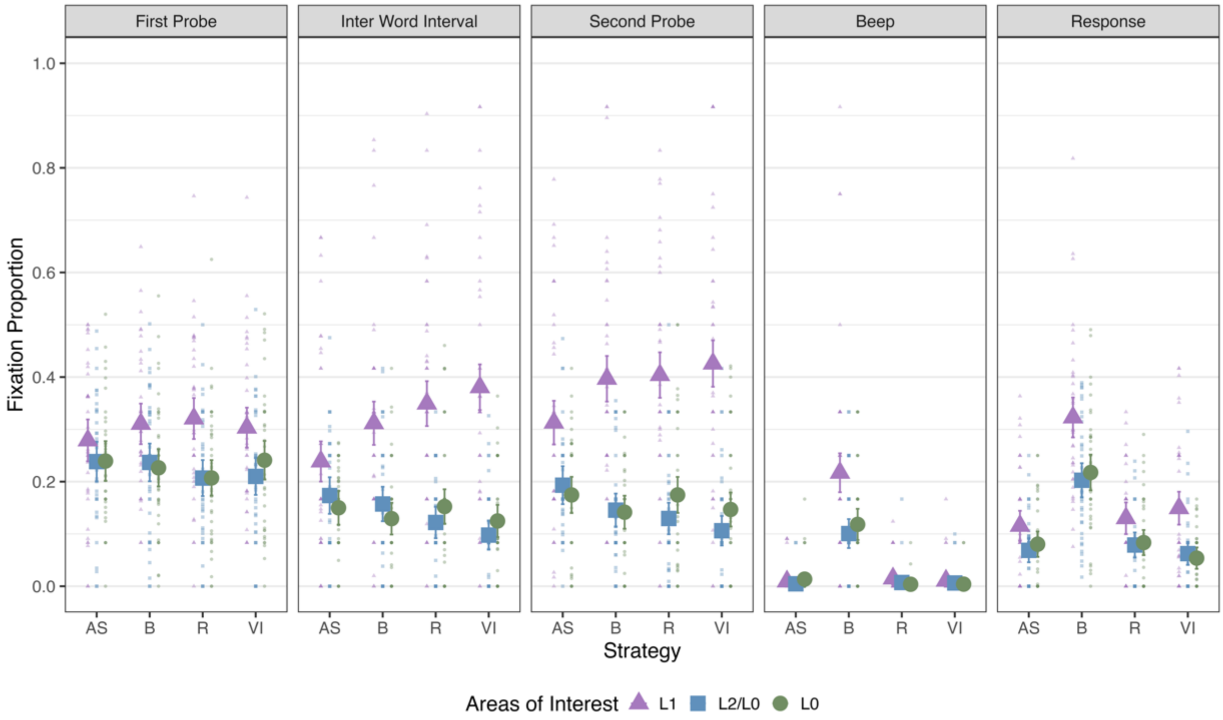

Figure S13

Fixation Proportion to the three AOIs across frames and strategies for LTM Test. The error bars indicate 95% within subject confidence intervals. AS: Articulatory Suppression, B: Baseline, R: Rehearsal, VI: Visual Imagery.

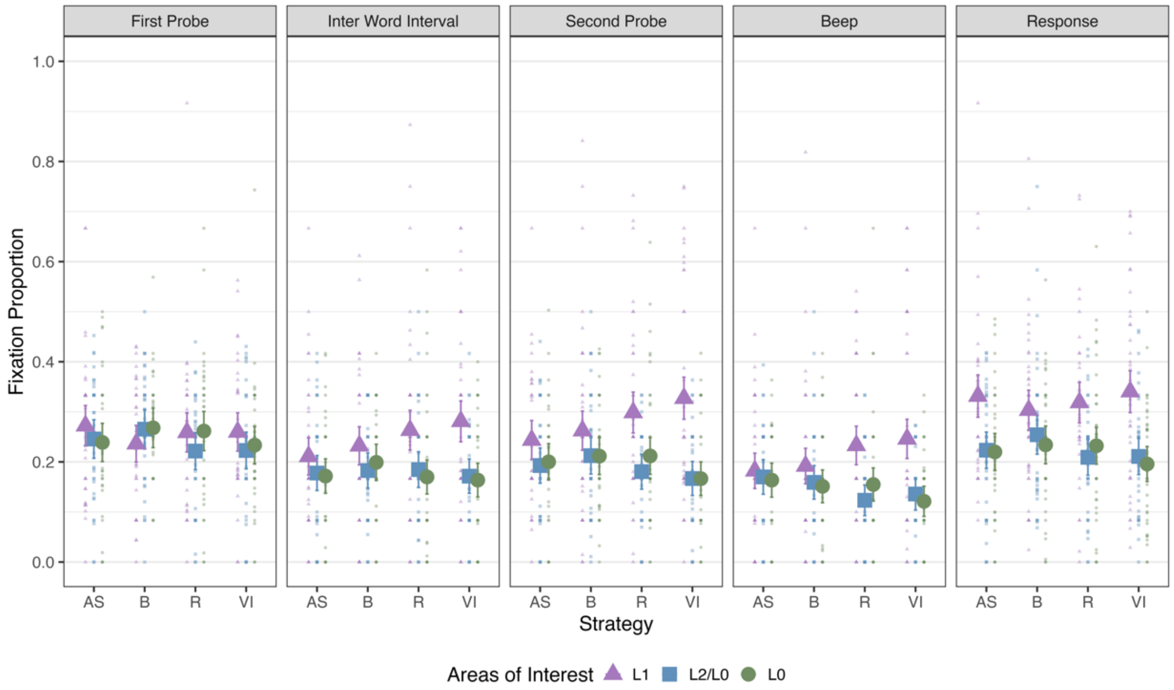

Table S7

Bayes Factor (BF10) for a main effect of strategy for comparison of fixation proportion to L1 across all frames.

Strategy	Baseline		Rehearsal		Visual Imagery	
	WM	LTM	WM	LTM	WM	LTM
			First Probe			
AS	0.06	0.16	0.06	0.06	0.06	0.07
Baseline			0.04	0.06	0.05	0.05
Rehearsal					0.05	0.04
			Inter Word Interval			
AS	0.44	0.05	1.77	0.09	16.2	0.22
Baseline			0.05	0.12	0.16	0.52
Rehearsal					0.07	0.06
			Second Probe			
AS	1.46	0.05	0.82	0.10	4.49	0.47
Baseline			0.04	0.07	0.05	0.46
Rehearsal					0.06	0.06
			Beep			
AS	1.4×10^5	0.05	0.30	0.11	0.41	0.38
Baseline			2.7×10^4	0.10	2.0×10^6	0.47
Rehearsal					0.29	0.06
			Response Screen			
AS	2.5×10^4	0.07	0.06	0.04	0.11	0.04
Baseline			7.9×10^3	0.05	1.5×10^3	0.06
Rehearsal					0.08	0.03

Similarly, in Experiment 2, for both PI and NoPI conditions, we descriptively observe higher LAN for Visual Imagery, Rehearsal and Baseline as compared to AS during the inter word interval and second probe, but these results are inconclusive. There is evidence against a main effect of strategy for the comparisons between Baseline, Rehearsal and Visual Imagery (Figure S14 and S15). Thus, a particular timepoint during the retrieval phase does not seem to influence whether one strategy shows higher LAN as compared to others, rather Baseline, Rehearsal and Visual Imagery show similar LAN throughout the retrieval phases of both the experiments.

Figure S14

Fixation Proportion to the three AOIs across frames and strategies for PI test. The error bars indicate 95% within subject confidence intervals. AS: Articulatory Suppression, B: Baseline, R: Rehearsal, VI: Visual Imagery.

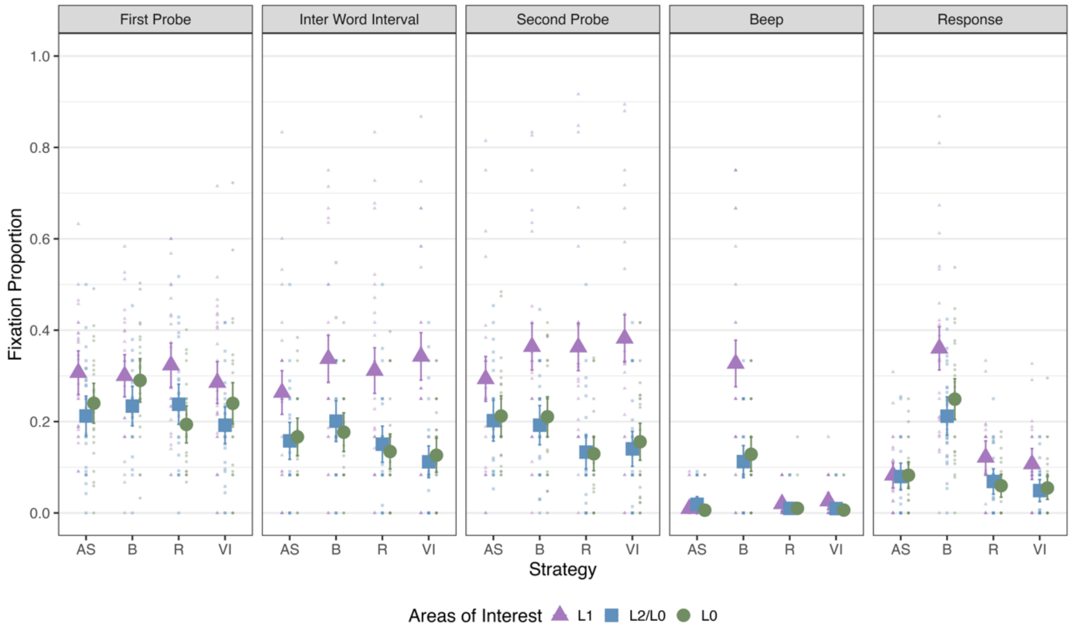

Figure S15

Fixation Proportion to the three AOIs across frames and strategies for NoPI test. The error bars indicate 95% within subject confidence intervals. AS: Articulatory Suppression, B: Baseline, R: Rehearsal, VI: Visual Imagery.

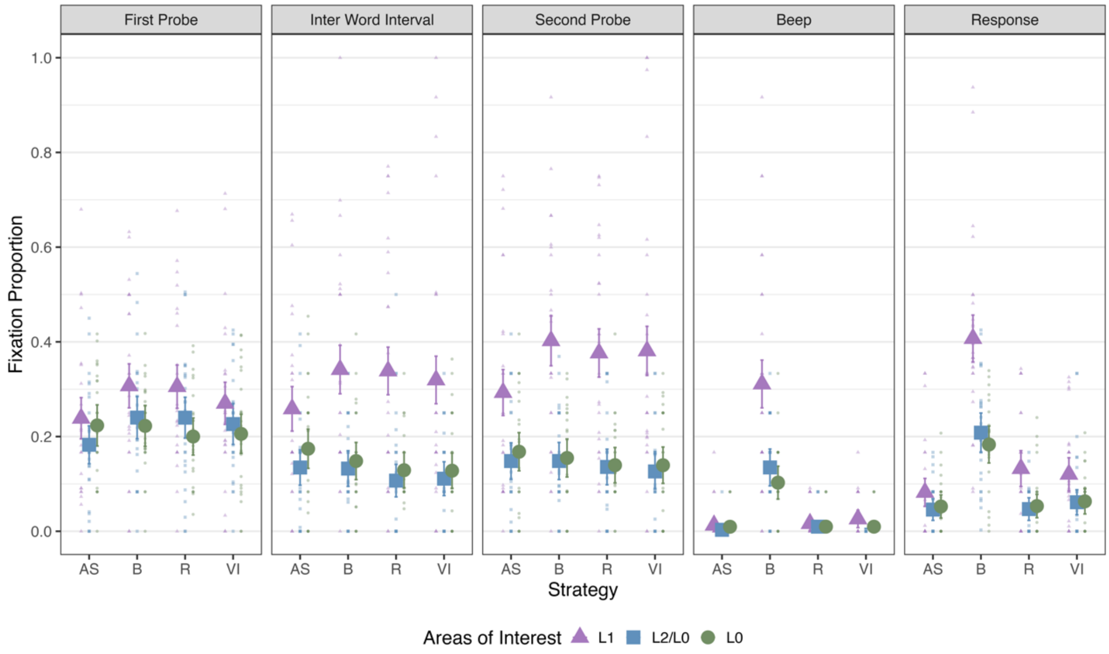

Table S8

Bayes Factor (BF10) for a main effect of strategy for comparison of fixation proportion to L1 across all frames.

Strategy	Baseline		Rehearsal		Visual Imagery	
	PI	NoPI	PI	NoPI	PI	NoPI
First Probe						
AS	0.05	0.06	0.05	0.47	0.06	0.05
Baseline			0.08	0.10	0.04	0.06
Rehearsal					0.09	0.42
Inter Word Interval						
AS	0.32	0.51	0.15	0.36	0.25	0.14
Baseline			0.06	0.06	0.05	0.06
Rehearsal					0.06	0.06
Second Probe						
AS	0.14	0.99	0.28	0.21	0.21	0.51
Baseline			0.07	0.08	0.05	0.06
Rehearsal					0.06	0.06
Beep						
AS	1.4×10^{7}	3.2×10^{6}	0.31	0.20	0.48	0.28
Baseline			8.7×10^{6}	8.9×10^{5}	3.0×10^{8}	2.6×10^{5}
Rehearsal					0.17	0.19
Response Screen						
AS	6.3×10^{3}	5.2×10^{4}	0.08	0.27	0.10	0.95
Baseline			1.8×10^{3}	5.9×10^{4}	1.3×10^{5}	6.0×10^{5}
Rehearsal					0.08	0.09

Figure S16

Fixation Proportion to L1 across all strategies and frames for WM test in Experiment 1. The error bars indicate 95% within subject confidence intervals.

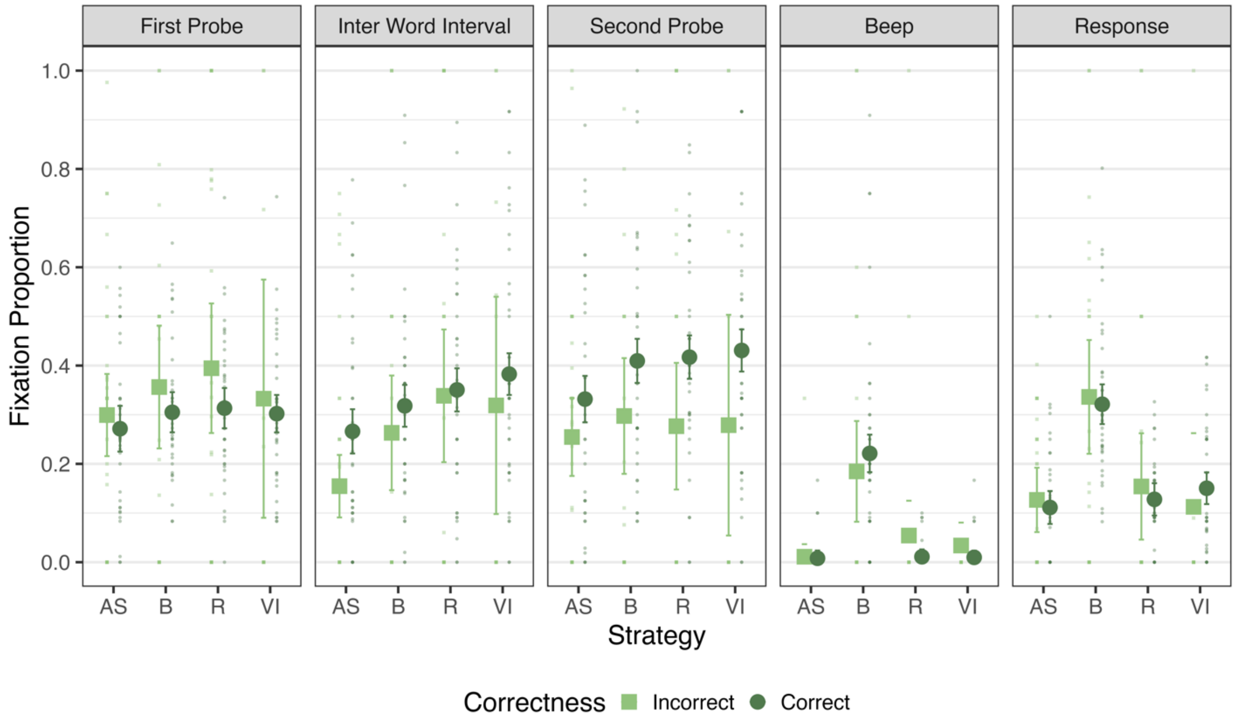

Table S8

Bayes Factor (BF10) for main effect of correctness across strategies and frames for the WM Test in Experiment 1.

Strategy	First Probe	Inter Word Interval	Second Probe	Beep	Response Screen
AS	0.05	0.63	0.10	0.22	0.06
Baseline	0.06	0.17	0.20	0.18	0.06
Rehearsal	0.07	0.08	0.19	0.38	0.08
Visual Imagery	0.08	0.12	0.31	0.39	0.17

5. Functionality of LAN across frames

The figures S16, S17, S18 and S19 display fixation proportion to L1 across all strategies and frames for both correct and incorrect trials. In the WM test, we observe a slight trend of higher fixation proportions for correct trials compared to incorrect trials during the second probe; however, there is evidence against a main effect of correctness (Figure S16 and Table S8). A similar trend is observed in the LTM test for rehearsal and visual imagery, and

there is evidence against a difference in fixation proportions between correct and incorrect trials (Figure S17).

Figure S17

Fixation Proportion to L1 across all frames and strategies for the LTM test for correct and incorrect trials. The error bars indicate 95% within subject confidence intervals.

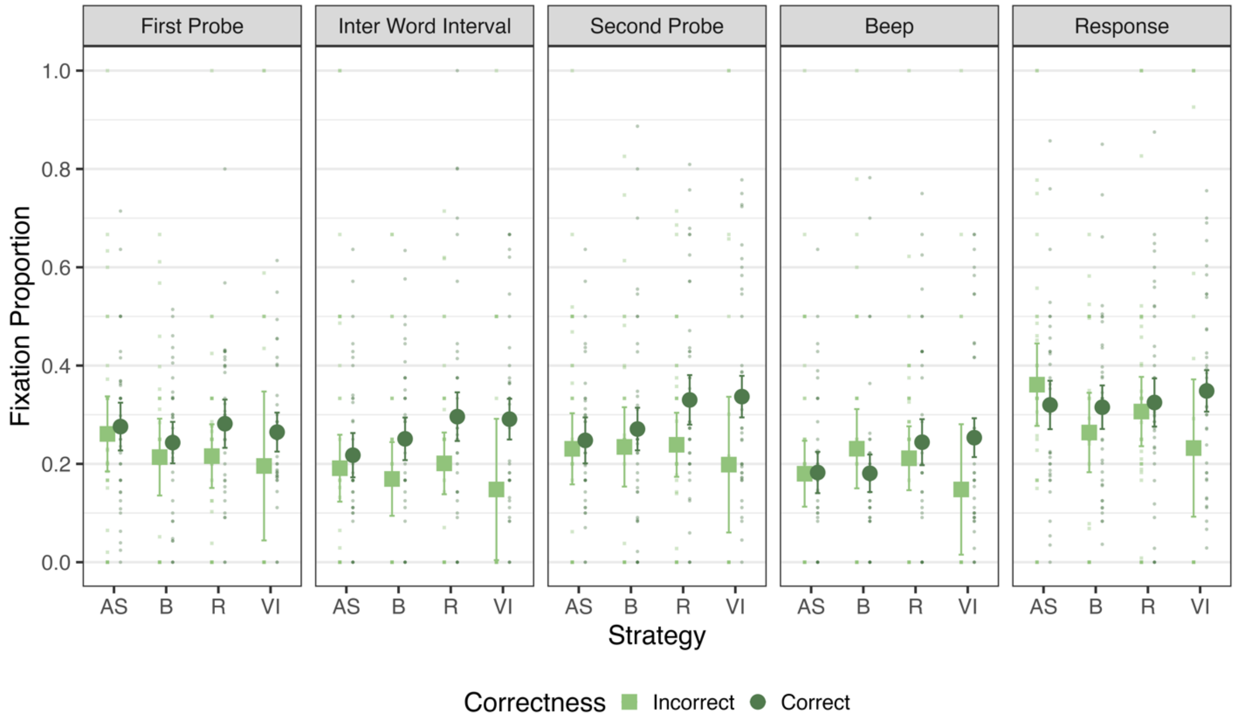

Table S9

Bayes Factor (BF10) for main effect of correctness across strategies and frames for the LTM Test in Experiment 1.

Strategy	First Probe	Inter Word Interval	Second Probe	Beep	Response Screen
AS	0.05	0.06	0.05	0.06	0.05
Baseline	0.07	0.46	0.14	0.07	0.18
Rehearsal	0.05	0.40	0.19	0.07	0.04
Visual Imagery	0.13	0.44	0.20	0.20	0.11

Similarly, in Experiment 2, for both PI and NoPI conditions, while there is a slight tendency for higher fixation proportions in correct trials, there is evidence against a meaningful difference (Figure S18 and S19). In the PI block, for visual imagery in Second

Probe and for Baseline during the Beep, we observe a higher LAN to L1. However, these results are inconclusive and in support of the main effect of correctness respectively. In this experiment, participants' WM performance may have been relatively high, resulting in fewer incorrect trials and a high proportion of correct responses. This could have made it more challenging to find conclusive evidence for the main effect of correctness. For the current study, a particular timepoint during the retrieval phase and the strategy does not seem to have an effect on the functionality of LAN across both the experiments.

Figure S18

Fixation Proportion to L1 across strategies and frames for correct and incorrect trials for PI condition. The error bars indicate 95% within subject confidence intervals.

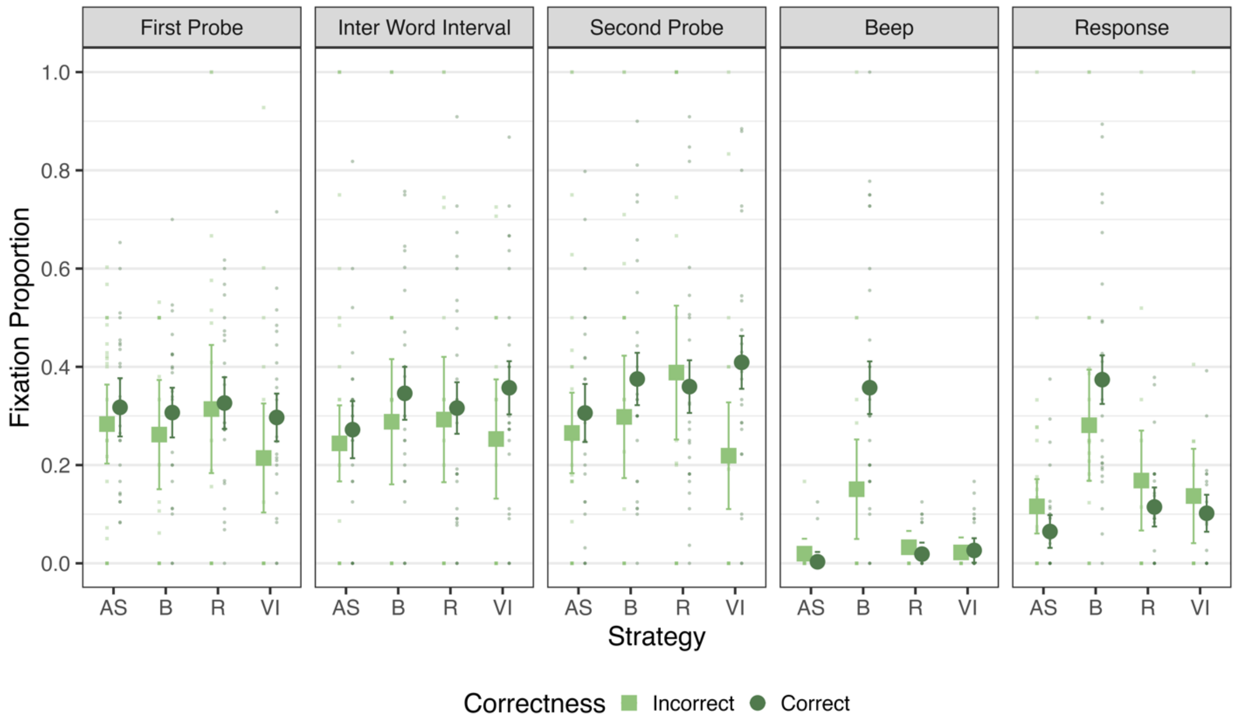

Table S10

Bayes Factor (BF10) for main effect of correctness across strategies and frames for the PI condition in Experiment 2.

Strategy	First Probe	Inter Word Interval	Second Probe	Beep	Response Screen
AS	0.05	0.08	0.06	1.42	0.14
Baseline	0.07	0.07	0.08	7.65	0.11
Rehearsal	0.09	0.14	0.07	0.22	0.17
Visual Imagery	0.08	0.29	0.98	0.54	0.11

Figure S19

Fixation Proportion to L1 across strategies and frames for correct and incorrect trials, NoPI condition. The error bars indicate 95% within subject confidence intervals.

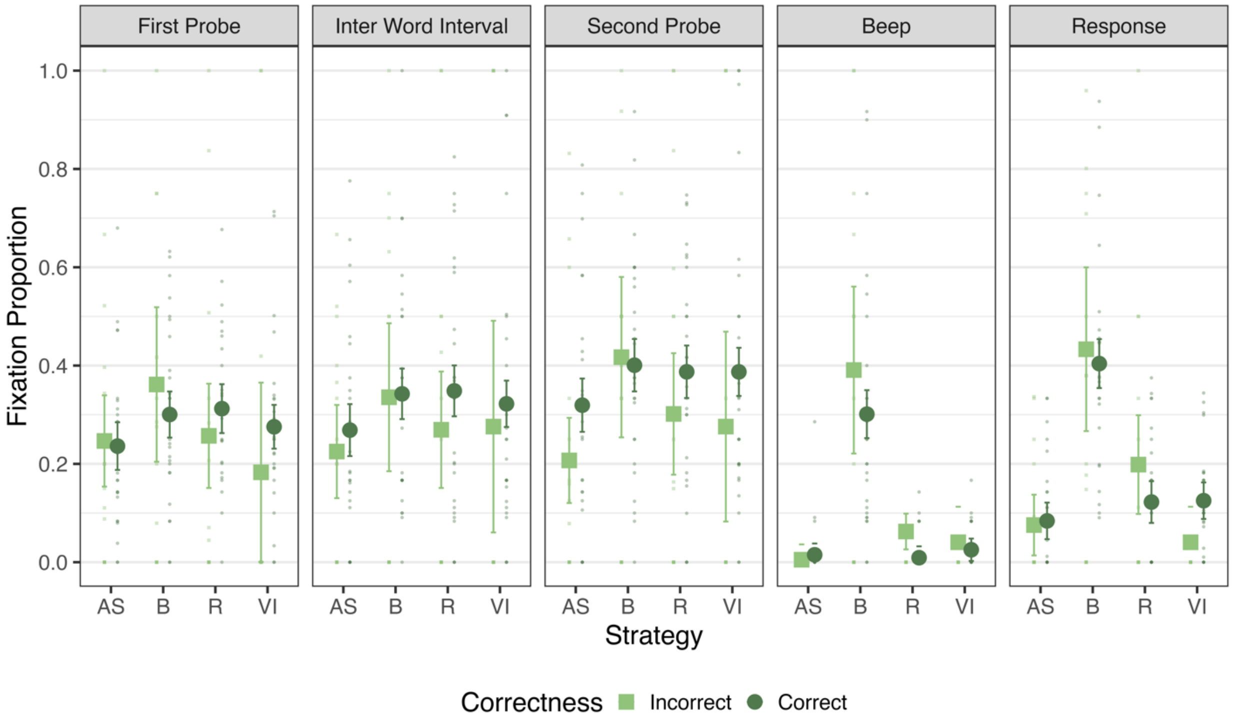

Table S11

Bayes Factor (BF10) for main effect of correctness across strategies and frames for the No PI condition in Experiment 2.

Strategy	First Probe	Inter Word Interval	Second Probe	Beep	Response Screen
AS	0.04	0.12	0.38	0.91	0.09
Baseline	0.06	0.10	0.07	0.88	0.06
Rehearsal	0.09	0.12	0.17	0.78	0.12
Visual Imagery	0.14	0.39	0.22	0.64	0.10

References

Bhanap, R., Oberauer, K., & Rosner, A. (2025, March 4). Investigating Retrieval Strategies in an Associative Recognition Test in Working Memory: Evidence from Eye Movements. https://doi.org/10.31234/osf.io/j34d6_v2

Bunting, M. (2006). Proactive interference and item similarity in working memory. *Journal of Experimental Psychology: Learning, Memory, and Cognition*, *32*(2), 183.

Kubinec, R. (2023). Ordered beta regression: a parsimonious, well-fitting model for continuous data with lower and upper bounds. *Political Analysis*, *31*(4), 519-536.

Nikolov, T. Y., Allen, R. J., Havelka, J., Darling, S., Van de Vegte, B., & Morey, C. C. (2025). Navigating the mind's eye: Understanding gaze shifts in visuospatial bootstrapping. *Quarterly Journal of Experimental Psychology*, *78*(2), 391-404.

Oberauer, K., & Bartsch, L. M. (2023). When Does Episodic Memory Contribute to Performance in Tests of Working Memory?. *Journal of Cognition*, *6*(1), 44.
